# Antibiofouling Coatings For Marine Sensors: Progress and Perspectives on Materials, Methods, Impacts, and Field Trial Studies

**DOI:** 10.1021/acssensors.4c02670

**Published:** 2025-03-05

**Authors:** Bichitra Nanda Sahoo, Peter James Thomas, Paul Thomas, Martin Møller Greve

**Affiliations:** † Nanophysics Group, Department of Physics and Technology, Allegaten 55, University of Bergen (UiB), 5007, Bergen, Norway; ‡ Measurement of Science Group, NORCE Norwegian Research Center AS, Nygårdsgaten 112, 5008, Bergen, Norway

**Keywords:** oceanographic sensors, water repellent, antibiofouling strategies, marine sustainability, environmental/economic impact, biofilm, fouling resistant coatings, fouling release coatings, biocide, field trial studies

## Abstract

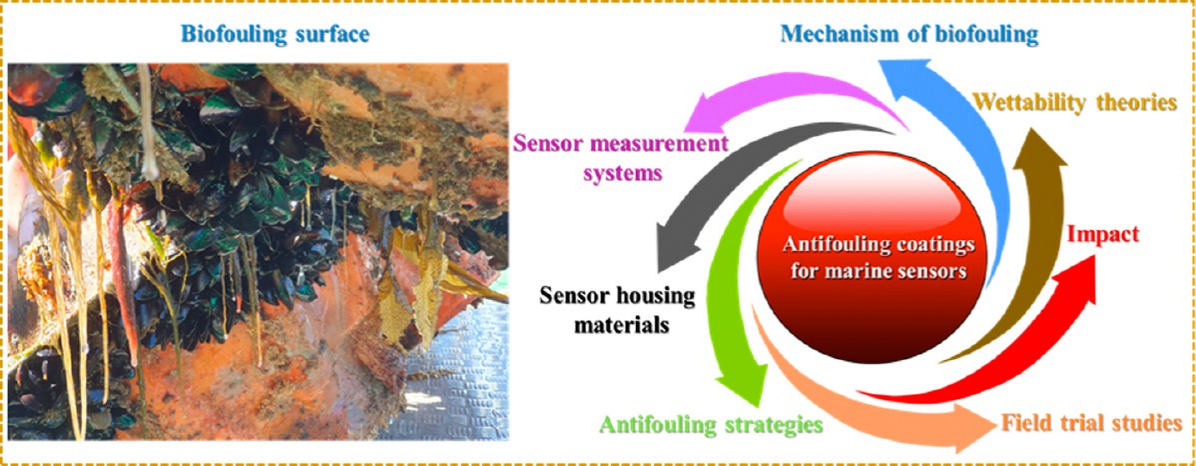

The attachment of marine organisms, for example, bacteria, proteins, inorganic molecules, and more on a sea-submerged surface is a global concern for marine industries as it controls the surface for further marine growth. Applications requiring the estimation of real-time information from oceanographic sensors conveyed for long-term deployment are vulnerable to biofouling. Therefore, an effective approach to controlling the biofouling that accumulates on marine sensors is paramount. To date, many technologies have been explored to impede biofouling; however, several factors constrain many strategies, including their reliance on environmentally toxic materials, high fabrication costs, poor coatings, and nontransparency. These challenges have motivated work to develop numerous advanced and innovative strategies based on mechanical methods, irradiation, and design of polymeric/nonpolymeric coatings with fouling resistance, fouling release, and fouling degrading coatings to protect marine sensors and housing materials from biofouling. This Review presents recent progress in the developed biofouling control strategies that have been applied to commercially available sensors and sensor housing materials. Moreover, recent findings in the literature are highlighted while considering the wettability principles for air and water environments, antifouling performance, practical feasibility, environmental and economic impact of coatings, and field trial studies. Here, we emphasize how these features can play major roles synergistically to affect antifouling coatings against nano- to microlevel organisms. This review will not only allow researchers to understand the design principles but also contribute to the development of new cost-effective strategies.

Continuous accumulation and growth of micro or macro-organisms on any seawater-submerged surfaces is called marine biofouling.^1,2^ The growth of marine organisms on submerged surfaces causes issues because it enhances the surface roughness, which further affects the lifetime of the materials,^3−5^ and also inhibits normal equipment function. Over the last decades, the rapid increase in marine exploration has enhanced the requirement for underwater optical and nonoptical instruments.^6^ One of the biggest concerns is the settlement and growth of microorganisms on the sensor surface and housing because of their disturbances in sensing characteristics, service life, sensitivity, data reliability, etc. During the formation of microorganisms on submerged surfaces, molecules, proteins, and cells first absorb onto the surfaces,^7^ subsequently triggering the settlement of microorganisms and the development of microfilms on the submerged surfaces.

The biofouling process proceeds through four distinct stages,^8^ as summarized in Figure 1a:

(i) A conditioning film is formed within the first few seconds after submersion of the substrate by attachment of seawater’s natural organisms.
(ii) In the second step, a primary film is generated by transporting the microbial cells to the surface. This happens during the next few hours.
(iii) After about a week, a microfilm is developed at the surface by attaching spores of macroalgae, protozoa, etc.
(iv) Finally, the microfilm promotes the adhesion of additional animal larvae, such as barnacles and mussels, which further assists in the formation of microfilm, as shown in Figure 1b. This phenomenon is called macrofouling or simply biofouling. Similarly, Figure 1c shows a photograph of a marine sensor before immersion in the seawater at Austevoll, Norway.

**Figure 1:**
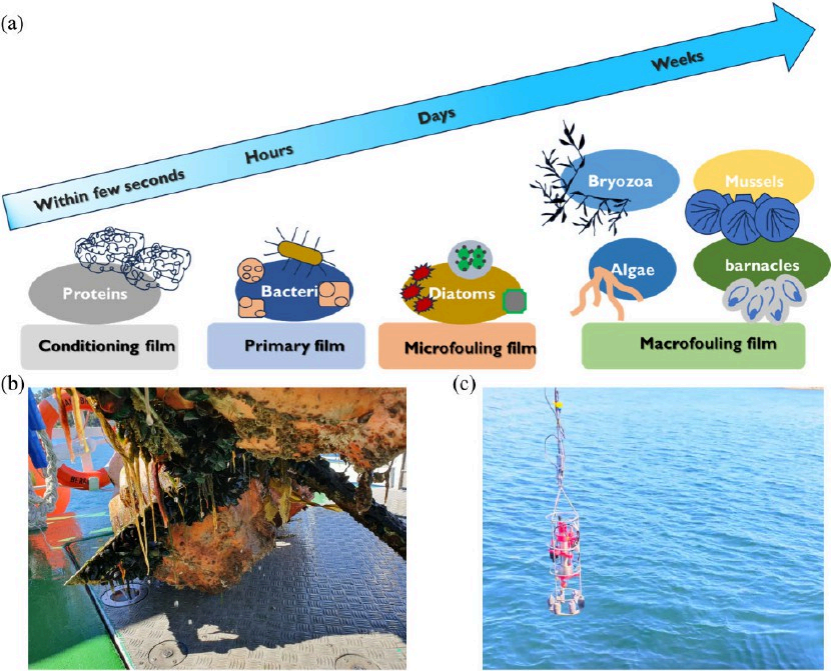
(a) Schematic illustration of the biofouling formation process at different stages, (b) fouling on the flotation device on the sea, and (c) photograph of the marine sensor just before immersing in seawater. Photographs are taken at Austevoll, Norway.

Over the past decade, sensor technology has gained more importance in the marine aquaculture industries.^9^ Furthermore, sensors are enhancing the precision fish farming approach by utilizing monitoring data to promote more environmentally sustainable practices in aquacultures.^10,11^ Similarly, marine research stations are established to collect data to regulate water quality along the coast, and most of these research stations are comprised of sophisticated oceanographic sensors.^12^ However, these sensors and their housing materials are prone to the settlement of marine microorganisms because they interact with biological and chemical processes in the seawater.^13^ Various strategies including physical and chemical antifouling processes have been demonstrated for marine fouling prevention and control to resolve this issue.^14−17^ Physical methods such as mechanical cleaning by wipers, spraying water jets, etc. are widely used but can only be used to remove foulants rather than block attachment. Although many techniques have been developed and employed in various harsh environments in the past decade very few of them have been sufficiently cost-effective to permit their application to commercial products.^18,19^

This Review aims to establish a connection between engineering, materials science, and biological materials. We will first review the surface wettability theories and topographic features of natural and artificial antifouling species including superhydrophilic, superhydrophobic, superamphiphobic, underwater oleophobic surfaces, and SLIP coatings, etc., determining the attachment behavior of microorganisms with the submerged surfaces (Supporting Information, S1). In the next section, we will briefly discuss the materials that are used for optical and nonoptical marine sensor instruments and their housing materials. Then, we describe the measurement principles of commonly encountered marine sensors, and a study is presented to discuss the effect of biofouling on marine sensors. Further, we highlight the recent antifouling strategies that have been carried out for combating biofouling of the marine immersed sensor surfaces. We then discuss the field trials that are carried out to understand the long-term effect of biofouling on oceanographic sensors. Then, we discuss the impact of biofouling phenomena on marine sustainability. Further, we have discussed marine industrial antifouling coatings for commercial and industrial elements. Finally, the conclusions and future aspects in this research field of ocean industries are also demonstrated.

## Wettability Principles for Antifouling Technologies

Before constructing the antifouling coating, it is necessary to understand factors that affect the creation of biofilm during the immersion of solids in seawater. It is well-known that at the nanosurface level, topographic features strongly influence surface wettability. Similarly, based on the accessible contact area of the surface, both the aspect ratio and topographic area of contact lead to the settlement of more organisms on the surface, and subsequently more force is required to remove them from the surface. Furthermore, the wettability of the surface influenced by water directly affects the antifouling or fouling release properties. The complexity of the coating materials, coating’s chemical composition, and coating’s surface free energy play key roles in the design of antibiofouling coatings. We have discussed the relationship between these parameters and surface wettability by demonstrating wettability theories and the wettability of natural and artificial bioinspired surfaces (see Supporting Information, S1).

## Marine Sensor and Housing Materials

Marine sensors are of global interest to monitor ecosystems and monitoring the efficiency and consequences of marine industrial activities.^20,21^ Underwater infrastructure is being constructed and operated for a variety of reasons, including oil and gas pipelines, coastal bases, and transshipment terminals.^21^ It is necessary to monitor these systems via measurement sensors to maintain a sustainable ecosystem, particularly in water areas of economic activity.^22^ The measurement of oxygen in oceanography is a large concern for marine scientists around the world. Typical parameters that are measured by sensors are oxygen, turbidity, pH, salinity, and so on. This is accomplished by a range of sensor technologies and measurement principles.

Table S1 demonstrates the list of materials that are used for sensor and sensor housing systems.^7,23,24^ Sensors are generally manufactured with one sensing/sensor area mounted in a sensor housing composed of some housing material. For any sensor immersed in seawater, choosing the appropriate material for device fabrication is crucial to ensure optimal sensor performance over a long period. Other important variables to consider are material availability, cost, and manufacturing capability. The use of metallic and nonmetallic materials for the design of marine-related devices in marine environments that are submerged or exposed to a highly corrosive saltwater ocean environment is a key concern.

### Effect of Biofouling on Sensors

Generally, biofilms start immediately on objects immersed in seawater. The sensor will be impacted in many ways including mechanical failure, microbial corrosion, and errors in recorded data or missing measurement data.^25−28^

**Figure 2:**
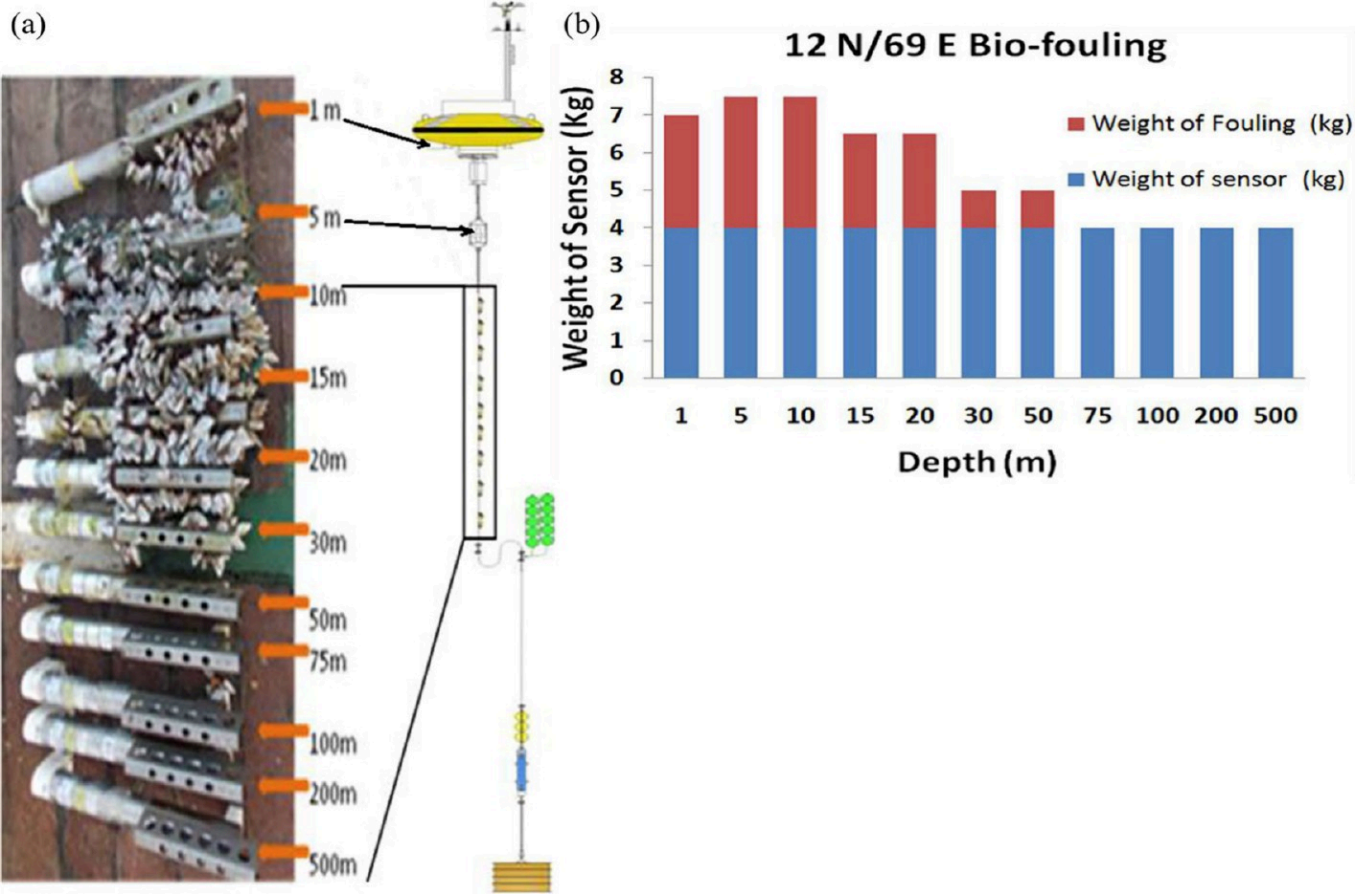
(a) Observation of biofouling growth at different depths. (b) Effect of the weight of fouling and the weight of the sensor at different depths. Reproduced with permission from ref (29). Copyright 2017 Elsevier.

Smith et al. demonstrated the influence of biofilm on gas membranes.^30^ It was found that thicker biofilms reduce the diffusion of gases through membranes, increasing the sensor’s response time. To demonstrate this research, Fraher and Clarke have reported the effect of biofilm on the concentration of dissolved oxygen (DO) level of the oxygen sensor.^31^ They have demonstrated that fouling caused by the accumulation of microorganisms on the membrane surface directly affects the movement of oxygen molecules to the electrode surface from the bulk of the molecules. Munro et al. studied the effect of biofilm on the response time for pH electrodes.^32^ They observed that the membrane attached to the biofilms enhances the response time of the pH electrode. It was found that increasing the thickness of the stagnant layer at the electrode surfaces led to an increase in the length of the diffusion path length for gas moving to the surface, which further increases the response time. The development of biofouling plays a major role in obtaining the accuracy of continuous real-time data collected by offshore moored buoy sensors.^33^ Venkatesan and his coauthors have carried out measurements using buoy-based sensor systems moored in the coastal waters and the northern Indian Ocean to study the effect of biofouling on sensors at different water depths.^29^ Biofouling was observed to be prevalent only at depths of up to 50 m, affecting the data in conductivity–temperature (CT) sensors positioned at varying depths in the Bay of Bengal and the Arabian Sea, as illustrated in Figure 2. Furthermore, Figure 2a,b demonstrates the physical observations and fouling statistics of the CT sensors at different depths following 202 days of offshore operation, respectively. The Fouling organisms consist of various species, making their separation challenging. Thus, it is important to understand the cause of metabolism behind these fouling organisms. For example, Zhang et al. reported the inhibition of marine biofouling by Butenolide via alteration of primary metabolism of three target marine organisms. They have studied the butenolides molecular targets in the three representative fouling organisms including in the Barnacle *Balanus Amphitrite*, the bryozoan *Bugula neritina*, in the bacterium *Vibrio* sp. UST020129-010.^34^ Furthermore, the metabolism of marine net pen fouling organisms’ community is studied by Ge et al. by conducting the study of NH_4_–N and PO_4_–P metabolism of fouling organisms which are associated with net pens.^35^ To study the metabolism of fouling organisms, they conducted field experiments for 64 days to study the biofouling process in Ailian Bay, China. It was observed that samples in the nets accumulated with fouling organisms. The excretion rate for NH_4_–N and PO_4_–P are 0.09 and 0.3 g/day, respectively. These nutrients may accelerate the primary production of fouling organisms. Additionally, Portas et al. demonstrated a multidisciplinary approach to investigate the characterization of biofouling by examining both fouling organisms and their metabolic outputs via analyzing the biochemical constituents of the biofilm matrix.^36^

Similarly, Koren and McGraw have reported the effect of analyte contraction at the biofilm–sensor interface on the sensor’s response time.^37^ They have reported that changes in the analyte concentration have a very low impact on the sensing performance of most environmental sensors. However, an analyte in the diffusion boundary layer (DBL) influences the sensor’s performance. In principle, if the biofilm controls the analyte concentration at the biofilm–sensor interface, then sensor performance is no longer affected.

## Marine Sensor Systems and Their Measurement

To date, numerous ideas have been proposed to monitor marine environment systems. The development of sensor instrumentation and its key role in marine environmental studies are considered to be the most innovative features in current oceanographic research. To study the effect of oceanic conditions on climate change, it is necessary to monitor the different parameters in seawater. Furthermore, important oceanographic measurement parameters such as pressure, salinity, turbidity, dissolved oxygen, sound speed, and density are required to be measured to study the effect of oceanographic conditions on climate change.^38^ In this section, we have reported an overview of the measurement principle used for monitoring parameters in the marine environment, in application areas such as monitoring the process of water quality, ocean environment, and marine fish farming.

### Principle of Measuring Parameters Monitored by Marine Sensors

Presently, the advancement of affordable sensors is crucial in marine environmental research and oceanographic studies. These types of sensors can be deployed at different platforms in the ocean environment. Generally, both physical and chemical sensors are used for monitoring different parameters such as salinity, turbidity, dissolved oxygen, temperature, pressure, pH, turbidity, etc. of the seawater.^39^ Furthermore, measurement accuracy, power consumption, resolution, etc. plays a major role in obtaining accuracy in the monitoring process.^40^

#### Pressure and Temperature Sensors

The main purpose of the measurement of pressure is to understand the inferring depth and vertical coordinates for other measurements.^41^ Most of the temperature sensors measure the ocean temperature ranges from −5 to 35 °C.^42^

**Figure 3:**
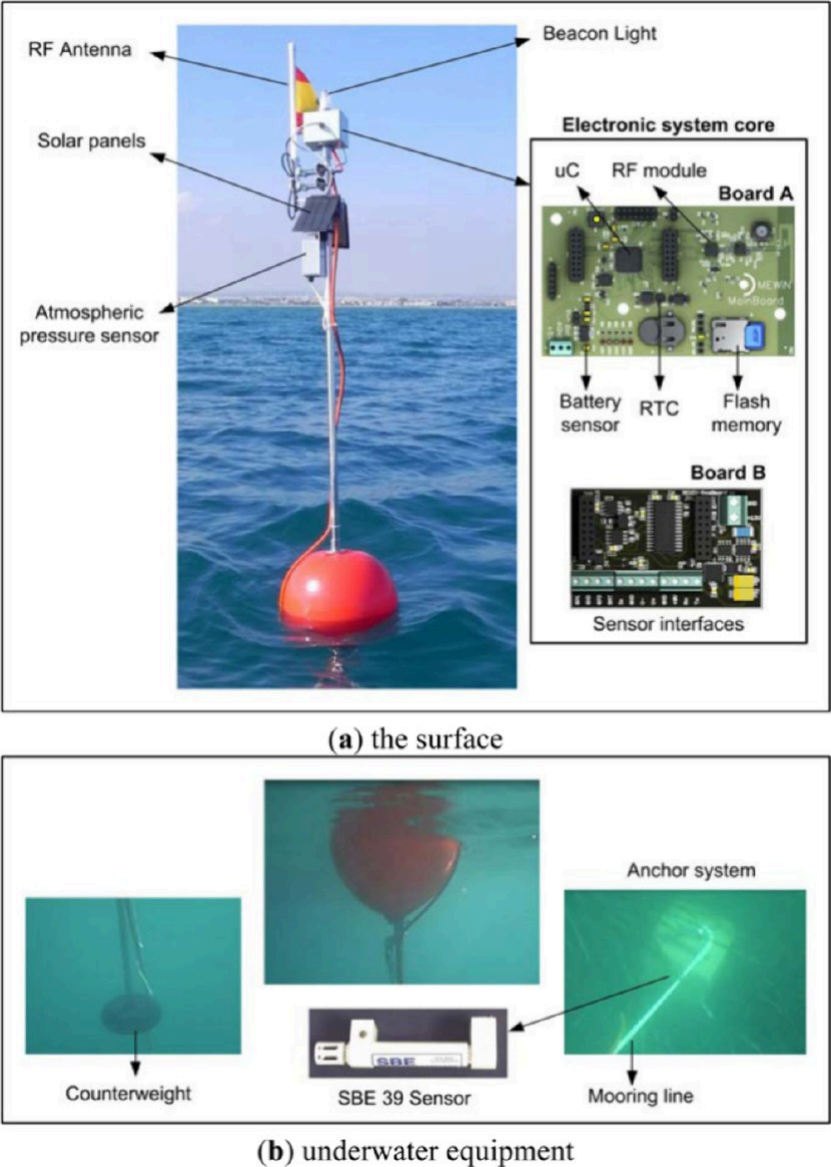
Typical components of an oceanographic sensor buoy. Reproduced with permission from ref (43). Copyright 2012 MDPI.

Temperature sensors such as electrical and optical sensors are mostly used in ocean measuring systems. In addition, thermistors, for example, SBE-911 have been also used for the measurement of temperature measurements. Albaladejo et al. demonstrated a new multisensor monitoring buoy system for application in a coastal shallow-water marine environment.^43^ Moreover, this buoying system measures environmental temperature, marine pressure, and atmospheric pressure in the aquatic environment using MCP9700, SBE 39 sensor, and YOUNG 61302L sensor, respectively. Figure 3 demonstrates the different components of an oceanographic buoy system that can be deployed at different heights in the seawater. However, the submerged part is very important because it gathered the data to the buoy and subsequently to the seabed, ensuring stability against marine currents and wind waves.

#### Conductivity and Salinity Sensor

**Figure 4:**
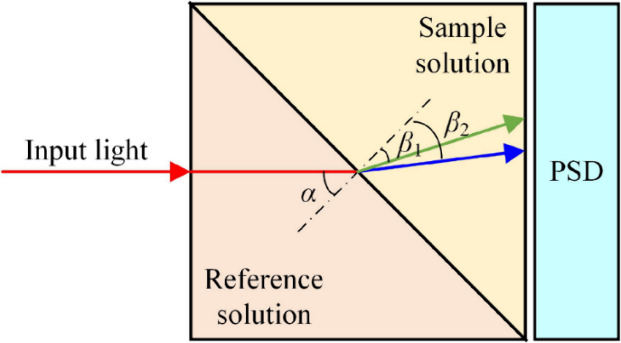
Illustration for the measurement of salinity using the beam deviation technique. Reproduced with permission from ref (46). Copyright 2022 MDPI.

**Figure 5:**
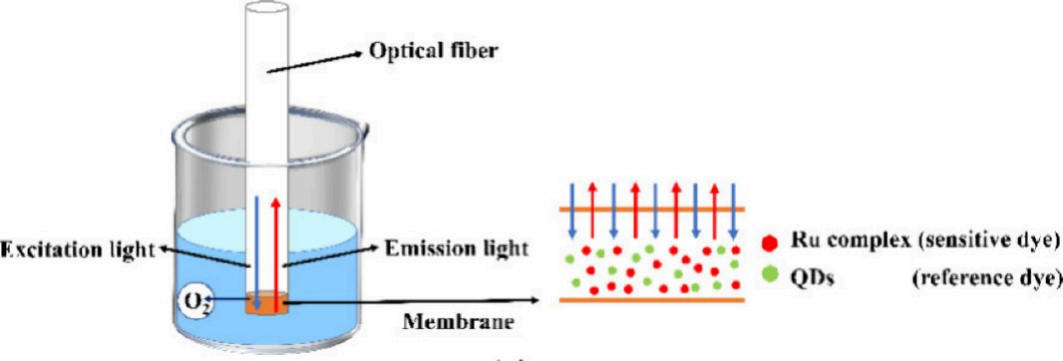
Illustration of the optical DO sensor. Reproduced with permission from ref (49). Copyright 2022 MDPI.

Generally, two types of sensors, such as electrodes and toroidal sensors, are used for oceanographic studies. It was found that electrode sensors contain 2 to 7 electrodes. On the other hand, inductive sensors are also used to measure the water’s ability to conduct an electrical current in terms of siemens per meter (S/m). In principle, the variation in salinity of seawater causes the ocean’s internal waves, which affects the stability of the ocean environment.^44^ Moreover, to conduct the salinity measurement, a CTD probe can also be used in hydrological observation because it works in the principle of electrical conductivity measurement.^45^ Furthermore, it is found that the measurement of salinity is mostly affected by the temperature in comparison to the pressure. Similarly, for example, CTD sensors such as thermohalimeters, which are mostly used for the measurement of salinity in oceanographic field.^46^ Moreover, the salinity can also be measured by using optical sensors. By measuring the refractive index in seawater, salinity can be measured by considering seawater as an optical medium. Generally, this type of measurement is done by using the beam deviation principle. As shown in Figure 4, a reference solution and sample solution are affected by an incident light.^46^ Both α and β are the incident and refraction angles, respectively, as shown in Figure 5. Based on the deviation of the beam propagation, a position-sensitivity detector (PSD) can be measured, which demonstrates the change in salinity.

In principle, both reflectance and transmittance types are considered while using the beam deviation method for the measurement of salinity. For example, Minato et al. demonstrated the transmittance-type refractometer for the measurement of salinity in 1989 transmission-type.^47^ To measure the salinity, they demonstrated a partitioned cell, which was comprised of two parts; one part contained seawater with a salinity of 35 and other part was filled with sample seawater.

#### Dissolved Oxygen (DO)

DO is an essential parameter that directly affects the marine environment’s sustainability. Thus, real-time monitoring of DO is required for all types of aquacultures.^48^ Nowadays, many optical sensors are developed on dynamic quenching principles. For example, Zhao and his coauthors demonstrated a DO sensor based on optical fiber, which works on the principle of dynamic quenching of fluorescence from a ruthenium complex molecule.^49^ They considered tris(4,7-diphenyl-1,10-phenanthrolin) ruthenium(II) dichloride complex (Ru(dpp)_3_^2+^) and CdSe/ZnS quantum dots (QDs) as an oxygen-sensitive dye, and a reference dye, respectively. Figure 5 shows a fiber-based DO sensor, where a ruthenium complex and Quantum DOTS (QD)s are demonstrated. When the optical fiber is excited with the incident light, a specific wavelength of light is absorbed by the fluorescent substances. It is worth noting that similar measurement principles have formed the basis of other oceanographic sensors such as those designed to measure CO_2_ concentration and NH_3_^49,50^ and pH.^51^

**Figure 6:**
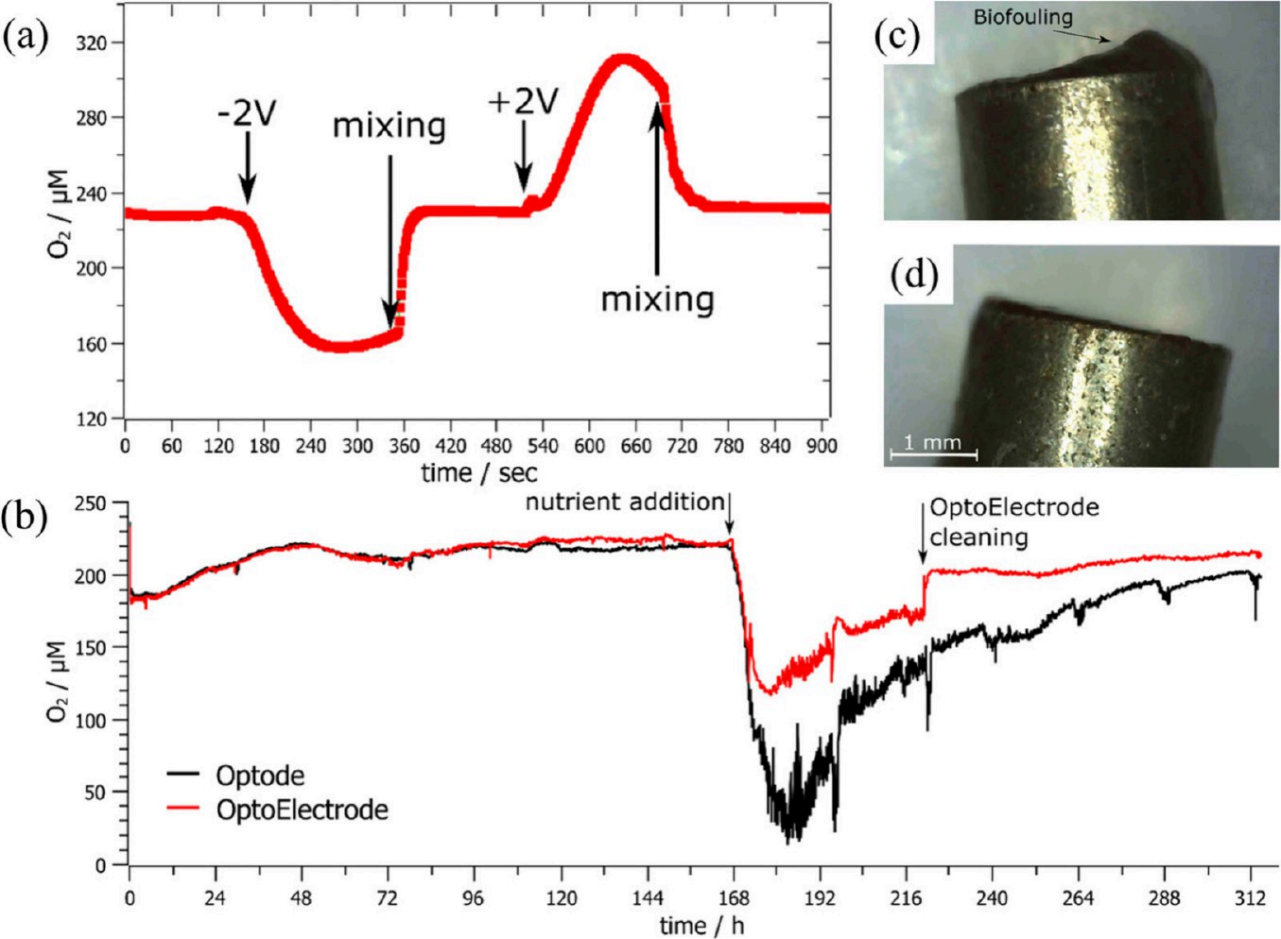
(a) Monitoring of oxygen level using optical sensor, (b) measurement of biofouling assay with and without addition nutrients for Optode and Optoelectrode, (c) images of optode with biofilm, and (d) image of OptoElectrode without biofilm. Reproduced with permission from ref (52). Copyright 2023 RSC Publishing.

Koren et al. have demonstrated the electrochemical effects that can be used to reduce the biofouling of commercial optical oxygen sensors (optode).^52^ This sensor works on the dynamic quenching principle. They introduced a water splitting process on the outer casting on the sensor, which results in increasing the pH and forming bubbles close to the optode surface. As shown in Figure 6a, when positive potential was applied, local O_2_ was increased. Further, local O_2_ levels were reduced when a negative potential was applied. To measure the biofouling assay, they immersed both unmodified optode and modified optode electrodes in the nutrient-enriched seawater. As shown in Figure 6b, they found that in the first 168h, the measured oxygen levels for both surfaces were similar. The optode electrode was polarized every 24 h, and the overall measured oxygen level was below 100%. This confirms the growth of planktonic bacteria in the system. Further, after 7 days, when more nutrients are supplied, the oxygen level is reduced drastically. However, the unmodified optode electrode shows a lower oxygen level due to the formation of biofilms compared with the modified electrode. Moreover, nutrient addition increases the possibility of the formation of a biofilm on both surfaces. Figure 6c and d reveal the formation of biofilm for both electrode surfaces. It was found that biofouling was not visible on the optoElectrode surface (Figure 6d). However, a thin biofilm was found with an optode (Figure 6c).

#### Turbidity

Measurement of the turbidity in water determines the optical properties of the water medium. In principle, water exhibits low light scattering and absorption due to the presence of particles including organic and inorganic clay, Algae, etc. As a result, it will enhance the turbidity of the water by increasing the light scattering and absorption. This measurement also determines the detection of the particles in water. A higher level of accuracy in the measurement of scattered light determines the low level of turbidity in the water. Generally, two main types of turbidimeters, such as an absorptiometer and a nephelometer, are used to monitor the turbidity of the water.^53−55^

## Recent Trends in Antifouling Strategies for Sensors

**Figure 7:**
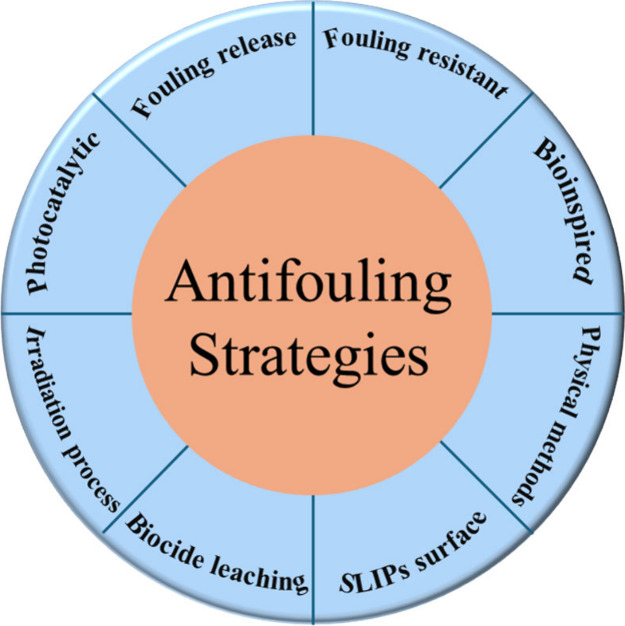
Eight major antifouling strategies.

Fouling affects sensitivity, limit of detection (LOD), selectivity, reproducibility, dynamic range, and lifetime of sensors.^56,57^ The key issue is here to balance the antifouling effect of sensors while not otherwise negatively impacting sensor operation. To prevent biofouling, first, we need to understand the purpose and reasons behind the protection of sensor and sensor housing materials against biofouling. Figure 7 has been used for the mitigation of biofouling for the marine submerged sensor surfaces. To overcome these problems, recent antifouling strategies have been improved in terms of materials and design to be compatible with different sensing-principles-based electrochemical sensors. Sensor surfaces coated with highly polar, hydrated chemical groups, water-soluble polymers, and materials containing cationic and anionic moieties have been shown to demonstrate a balance between maintaining normal sensing activity and antifouling property.^58^ In addition, other strategies are employed to improve the antibiofouling property of sensors without disturbing the sensing principle of sensors. Irrespective of the sensing principle of different sensors, most mitigation strategies are divided into two categories such as active and passive, which use physical, chemical, and biological approaches.^59^ For example, physical strategies in which the surface is not required to be changed instead of a cover material with a layer can be used for components that reduce direct contact between the foulants and electrode. BSA (bovine serum albumin) molecules are mostly used in these strategies. Another surface modification can be done using the mechanical coating method, where the antifouling coating can be formed using physical methods such as dipping, spin coating, spray coating, etc.^59^ Additionally, bioinspired surfaces such as superhydrophobic surfaces can be used to reduce the antibiofouling property of the surface without affecting the measurement principle of the sensors. Furthermore, the use of nanostructured electrodes (NSEs) minimizes the impact of surface fouling and increases the sensing activity. Nanostructured electrodes with surface features, for example, pores with a size of 1–100 nm, can be developed using etching, annealing, oxidation, and reduction process. These features prevent the attachment of unwanted materials on the electrode surface and minimize the loss of sensitivity of sensors.^60^ Furthermore, functional modification can be done to the electrode surface to increase both antifouling properties and sensing properties, for example using alkaline thiol, self-assembled monolayers, etc.^61,62^

**Figure 8:**
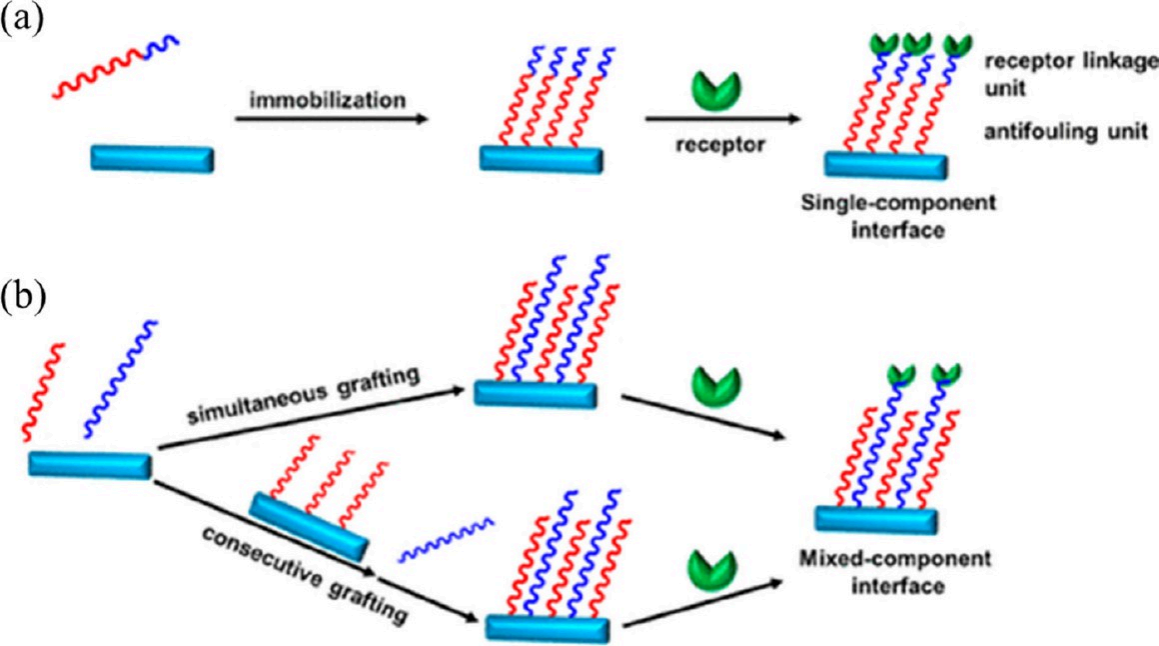
Fabrication principle of antifouling sensor. (a) The red color demonstrates for antifouling unit, and the blue color demonstrates the bioreceptor units. (b) The construction of a heterogeneous sensory interface using antifouling and anchor units via the grafting approach. Reproduced with permission from ref (139). Copyright 2020 American Chemical Society.

Incorporation of receptive elements into the sensory interface can be done to balance the antifouling and sensing performance of the sensors. For example, the incorporation of functional biorecognition elements into the sensory surface can be implemented to examine the antifouling performance of the surface. As shown in Figure 8a, materials including CB (carboxybetaine), peptides or functional group terminated OEGs,^63^ can modify the interface in one step. Similarly, as shown in Figure 8b, an extra anchor component can be fixed either at the same time or sequentially to create a mixed interface, in which case the antifouling component is not easily functionalized. This process can be easily achieved by self-assembly, electro-grafting, or copolymerization. In principle, if nonfouling elements are coimmobilized onto their surface in an appropriate ratio, this can provide the best sensor performance in terms of antifouling performance without impacting to receptor of the sensor.

### Physical Biofouling Reduction Strategies

Physical strategies are mostly used to hinder or delay the attachment of fouling organisms to surfaces or to facilitate the removal process of foulants. This technique encompasses various methods for physically cleaning surfaces, ranging from basic mechanical cleaning to advanced removal process.^64^ Some alternative approaches also work on the principle of roughened surface engineering devices or their wetting properties to block foulant adsorption.^65^ These approaches act as effective antifouling techniques, although they exhibit slow response time and demonstrate restricted mass transport kinetics, etc.

#### Wiper Technologies

One effective and applied method for cleaning fouling on sensors is the use of wiper technology (this is used for many sensors, e.g., Aanderaa instruments). However, these techniques are not acceptable methods for cleaning all types of sensors. Thus, the exact cleaning strategies used should be considered during the design of the sensor. Wiper technologies such as mechanical washing using brushes or water jets are useful for combating fouling phenomena during or after the deployment of sensors. Furthermore, waterproof ports, shafts, connections, and metallic components are prone to corrosion during the deployment of instruments in the ocean. Thus, protection from corrosion and biofouling, a proper cleaning strategy, or antibiofouling coating will be required for the sensors.^66^ A sensor device developed by the U.S. Navy vibrates when excitation with electric pulses removes fouling materials. Similarly, a marine sensor can be mounted in a protective house and can be immersed in water for a specific period to obtain for the collection of data.^67^ In this approach, a protective shutter can be used to facilitate the sensor surface for the measurement of data and prevention of the fouling process.^68^

#### Using Open Systems

Open systems have been an effective approach for cleaning foulants on marine sensors. It was demonstrated that marine sensors such as fluorometers and transmissometers (considered open systems) installed on the east coast of the United States in 1988 were maintained according to the principle of open system.^69^ Also, research indicated that an automatic scrubbing mechanism developed for a fluorometer can effectively clean the optical window once daily. To mitigate the biofouling of moored transmissometers, a transparent coating (Aquatic) was used to paint the optical windows before they were used. However, a biofilm was formed with optical windows, although it demonstrated the biofouling inhibition on the transmissometer at the East Coast.^69^

**Figure 9:**
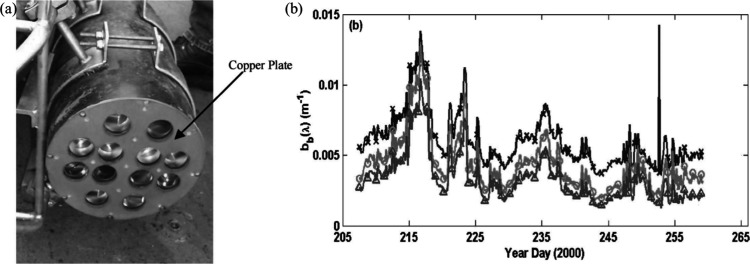
(a) Image of sensor Hydroscat-6 incorporated with cupronickel and (b) backscattering data collected by Hydroscat-6 in three wavelengths of 442, 510, and 620 nm within 60 days of deployment. Reproduced with permission from ref (70). Copyright 2004 American Meteorological Society.

Similarly, to reduce the biofouling on moored transmissometers, bronze rings incorporated with tributyl tin oxide were demonstrated by Butman and Folger.^71^ Alongside Butman and Folger’s approach, Strahle et al. utilized a comparable methodology that utilized porous plastic antifouling rings for the development of antifouling coatings.^72^ Similarly, Hobi Laboratories developed a cupronickel plate to inhibit the biofouling process on their backscattered sensor, Hydroscat-6, as shown in Figure 9a.^70^ As shown in Figure 9a, it is demonstrated that due to direct contact between two different metals, electrolysis around the sensor ports was avoided by electrically isolating the copper plate from the anodized aluminum pressure box. This method increases the fouling resistance of the sensor. It was found that there was no discernible biological development on the sensor ports during its 60 years of installation in coastal waters (Figure 9b).

#### Using Closed Systems

It has been observed that closed systems have an advantage in mitigating biofouling compared to open systems. For closed instruments, samples are not exposed to sunlight inhibits the photosynthesis process.^70^ For example, spectral AC meters are closed systems, but they are vulnerable to the attachment of biofouling organisms. Devis and his co-workers created a chemical technique to lessen the effects of biofouling for one six-wavelength (440, 540, 600, 650, 676, and 694 nm) AC meter (placed at 11 m depth) and two three-wavelength (650, 676, and 710 nm) AC meters (placed at depths of 9 and 40 m).^73^ From March 1993 to September 1993, all of the instruments were in the Bering Sea. They employed this technique by using a bromine solution in the instrument tubes via an outer-vented canister that contained solid bromine tablets in an inner-perforated canister. Prior to measurements, the tube was thoroughly cleaned with seawater to prevent contamination of the bromine solution.

**Figure 10:**
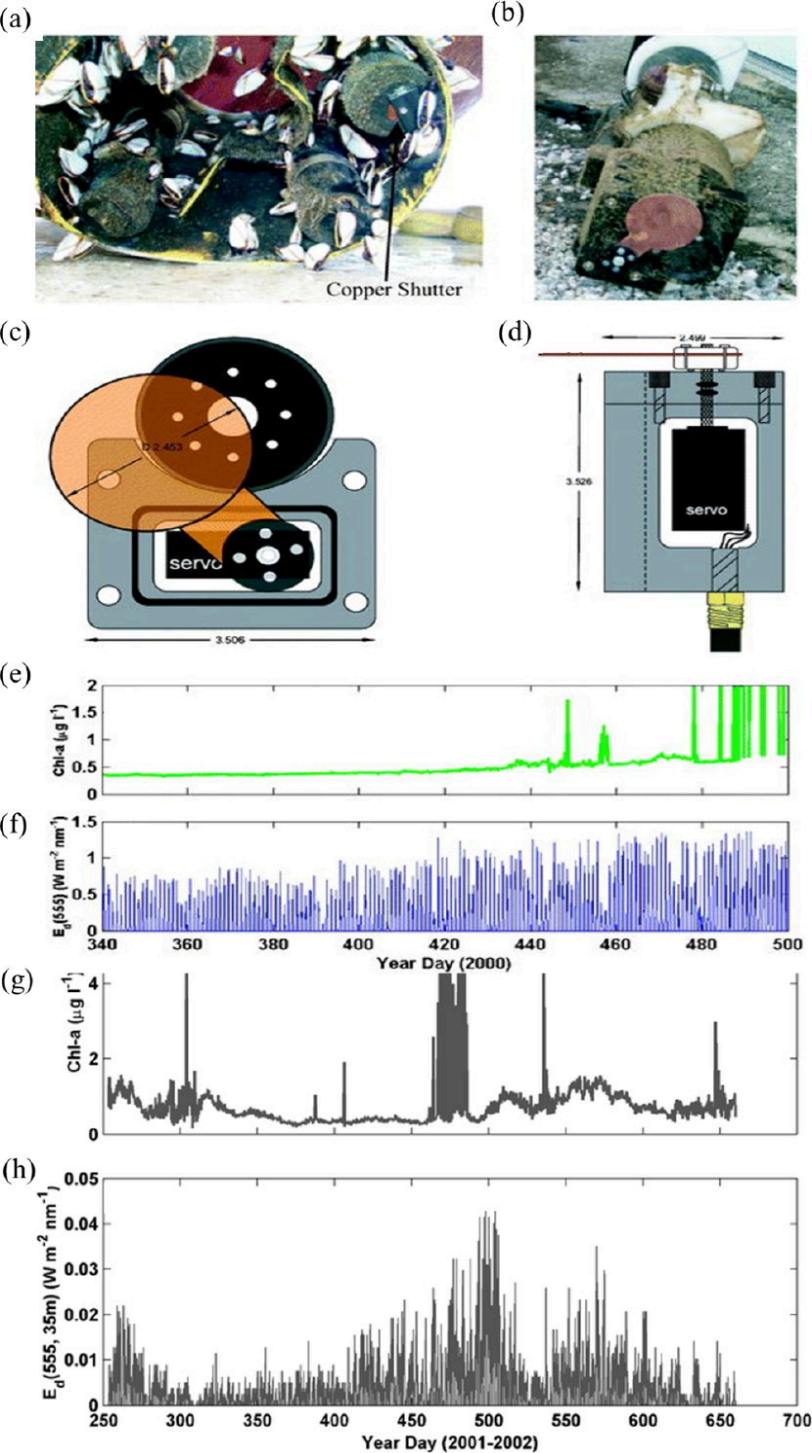
(a) Copper-shuttered ECO fluorometer, (b) copper-shuttered radiometer, (c) top view of copper-shuttered radiometer, (d) side view of the copper-shuttered radiometer, (e) chlorophyll fluorescence data collected by the ECO fluorometer, (f) data collected by radiometer, (g) average chlorophyll concentration monitored by ECO fluorometer, and (h) irradiation measured by the radiometer. Both sensors are deployed in the Japan Sea from Sept 2001 to Oct 2002. Reproduced with permission from ref (70). Copyright 2004 American Meteorological Society.

Similarly, shutter systems have been considered as suitable antifouling strategies for marine sensors to combat fouling organisms. Furthermore, Chavez et al. introduced a copper shutter mechanism for spectroradiometer.^74^ Copper-shuttered ECO fluorometer and radiometer were demonstrated by Manov et al., which were based on the shutter principles shown in Figure 10a,b. This shutter system is developed on commercial high torque servo power battery technology. Figure 10c illustrates the copper plate connected to the servo through a waterproof dynamic O ring seal, and Figure 10d demonstrates a cable connecting the microcontroller, datalogger, and battery backup to the servo. Furthermore, until the conclusion of the measurement process, the copper shutter remains closed over the spectral radiometer. In principle, optical VSF is used by oceanographers to predict light propagation, image degradation, Oceano color biological environment, etc. On December 5, 1999, ECO fluorometers were placed in the Sargasso Sea at a depth of two meters. At depths of 7 and 20 m, copper-shuttered spectral radiometer systems were also used. All these systems were successfully restored with antifouling data after almost five months. It was found that the fluorescence data were mildly biofouled (level 1 biofouling) up until day 490, as illustrated in Figure 10e,f. It was found that after 490 days, data is heavily biofouled. Furthermore, optical data for a longer duration time obtained by using the copper-based antifoulant method are demonstrated in Figure 10g,h. Copper shutters were used along with a spectroradiometer and ECO fluorometer before their deployment in the North Pacific Ocean off the coast of Japan, in 2001 September for 410 days year until 2002 October. Upon recovery of these instruments, little or no biofouling was found, which is revealed in Figure 10g. On the other hand, fluorometer shows O biofouling. However, the decrease in irradiation and radiation reduces the biofouling process in 410 days due to the cloud formation over the region, as shown in Figure 10h.

### Irradiation Techniques

To prevent antifouling on marine sensors, irradiation processes using ultraviolet light, laser, X-rays, gamma ray irradiation, and ultrasonics have been considered. However, the main challenge of these methods is the potential for the photobleaching of any compounds after irradiation.

#### UV Radiation

UV irradiation is a new technology that is employed for biofouling control on ship hulls and marine sensors. UV rays in the wavelength range 100–400 nm are used for antifouling strategies, acting to kill microorganisms by destroying the DNA of the organisms.^75^ Titus et al. demonstrated the protection of marine sensors and other submerged surfaces from marine biofouling using UV light.^76^ This new method has been confirmed in the protection of sensor surfaces from any biofouling. It was found that distribution of the UV–C light emitted from UV-LEDs, was achieved using silicone light guides.^77,78^ Individual LEDs can be otherwise configured in such a way that their emitted light irradiates the whole sensor surface.^79^ Furthermore, Lakretz et al. demonstrated the process of controlling the suspended planktonic cells in water using various UVC wavelengths and doses.^80^ They used UV wavelengths between 220 to 280 nm. They found that wavelengths between 254 to 270 nm performed well in the bacterial inactivation process

### Pulsed Laser (Non-UV) Radiation

Another anti biofouling strategy such as laser irradiation for the prevention of biofouling by barnacles and diatoms is recently demonstrated. Nandakumar and his coauthors performed the antibiofouling efficiency using pulsed laser irradiation studies on diatoms, such as Skeletonema costatum and Chaetoceros gracilis.^81^ Both the duration of exposing light and laser energy enhance the mortality of the film. Further Nandakumar and his coauthors have also investigated the fouling process that controlled laser irradiation technique.^82^ They used the Nd:YAG (neodymium doped yttrium aluminum garnet) laser (Spectra-Physics Nd:YAG GCR-170, 0.1 j/cm^2^) to perform the experiments. To carry out the laser irradiation experiments, they have used pulsed irradiation with a wavelength of 532 nm (green light), a peak power of 20 mW, a pulse width of 5 ns, and a repetition rate of 10 Hz. It was found that mortality observed among two diatom species was enhanced with an increasing laser radiation time. Also, the number of diatoms in the samples demonstrated that the difference in cell counts compared to the control group decreased over time. On the other hand, damage to the planktonic diatoms can occur due to the irradiation of low pulsed power. As a result, this process can be considered a leading technique to combat biofouling.

Similarly, Stanislav and their coauthors investigated the antibiofouling study using laser radiation with wavelengths of blue (448 nm) and infrared (1016 nm) for two different commercial antifouling coats.^83^ It was revealed that when the coating reflects more light, it absorbs less. This indicates that it uses less laser power, works more efficiently, and does not damage the coating. Furthermore, Thomas and Vasa demonstrated the analysis of biofouling issue by using the laser-induced breakdown spectroscopy technique (LIBS).^84^ They created a LIBs database for certain types of algae, like *Nitzschia sigma* and *Chaetoslorenzianus*, and bacterial species such as *Pseudomonas aeruginosa*, *Bacillus subtilis*, and *E. coli*. They also found that the strength of LIBS signals got stronger as the biofilm grew on the surface.

#### Ultrasonic Radiation

The applications of ultrasonic irradiation methods to control biofouling have been investigated. Ultrasound irradiation has been shown to prevent cyprid settlement on submerged surfaces.

**Figure 11:**
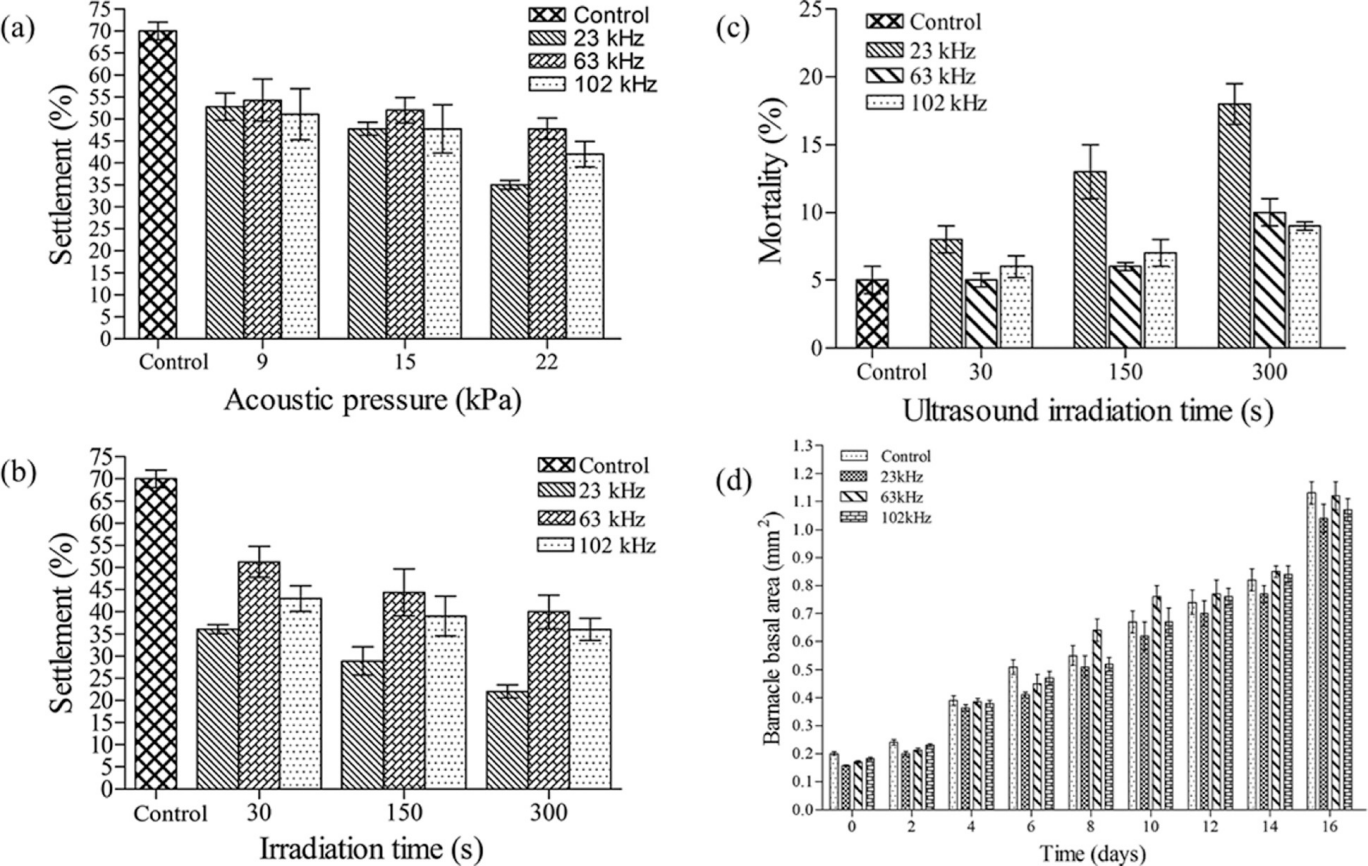
Cyprid settlement vs ultrasound exposure: (a) acoustic pressure vs settlement for 30 s, (b) irradiation time vs settlement, (c) ultrasound irradiation time vs mortality of cyprids at 20 kPa, (d) berancle of the basal area vs time (days) at different ultrasonic frequencies. Reproduced with permission from ref (85). Copyright 2011 Taylor and Francis.

Ultrasonic wave irradiation at a high frequency has been shown to have a mortality effect on fouling organisms such as barnacles, mussels, etc. To date, several studies on combating biofouling using the ultrasonication method have been reported.^85−89^ It was observed that high-intensity ultrasound creates strong liquid shear forces during the ultrasonic irradiation process, which stops from settling on the submerged surfaces.^85^ Its impact on barnacle settlement, however, has not been studied so far, thus, Kitamura and his co-workers further investigated the effect of ultrasonic waves on the survival of laboratory-reared *Balanus Amphitrite nauplii*, at three frequencies such as 19.5, 28.0, and 50.0 kHz.^86^ Among these three frequencies of ultrasonic waves tested, waves at 19.5 kHz showed the greatest effectiveness in reducing the survival rate of barnacle nauplii. Further, to prevent the settlement of cyprid, many methods have been employed by many researchers. For example, Guo and their coauthors performed antifouling testing by evaluating settlement inhibition of barnacles (Amphibalanus amphitrite) and cypris larvae exposed to ultrasound for periods of 30, 150, and 300 s at three different acoustic pressure levels of 9, 15, and 22 kPa and at three different frequencies of 23, 63, and 102 kHz. It was discovered that 23 kHz produced the highest cyprid mortality and the lowest settlement.^85^ The frequency that caused the highest cyprid mortality and the least amount of settlement was 23 kHz. As illustrated in Figure 11, the cyprid settlement was 2 times lower after 30 s of exposure to 23 kHz at 22 kPa. Likewise, the impact of using low-frequency sound to stop the settlement of zebra mussels on submerged structures was studied by Donsky and Ludyanskiy.^90^ They found that zebra mussels and larvae were damaged under the influence of sound and vibration, preventing the settlement and growth onto exposed surfaces. Although this strategy demonstrated excellent performance, protecting large surfaces like boat hulls from biofouling and battery-powered sensors brings more challenges at present. Additionally, Mott et al.^91^ examined how to control the formation of biofilm on glass tubing and by using ultrasonic irradiation. They used axially propagating ultrasound (APU) to remove mineralized *Proteus mirabilis* biofilms from water-filled glass tubes. Biofilm was removed from a 15 cm glass tube between 0 and 120 s using the cavitation activity generated by an APU operating at 150 kHz. According to their research, two 30 s APU pulses at 150 kHz removed 54.8% of the biofilm from 7 cm tubes. This was similar to the 60.9% removal that was obtained by sonication at 33 kHz in a traditional sonic cleaning bath. At the far end of the 50 cm tubes was found to effectively remove 40% of the biofilms at 350 kHz, 70% at 150 kHz and 90% at 20 kHz.

### Chemical Strategies

Currently, marine sensor manufacturers rely on both active and passive technologies to combat marine fouling phenomena. Active technologies based on mechanical tools such as wipers or scrapers, bubble blasting,^92^ or UV irradiation^93^ prevent settlement on sensor surfaces. However, the majority of passive antifouling systems rely on the utilization of biocides, especially copper, which has been used to design antifouling coatings for both sensor surfaces and their housing.

#### Fouling-Resistant Coatings

**Figure 12:**
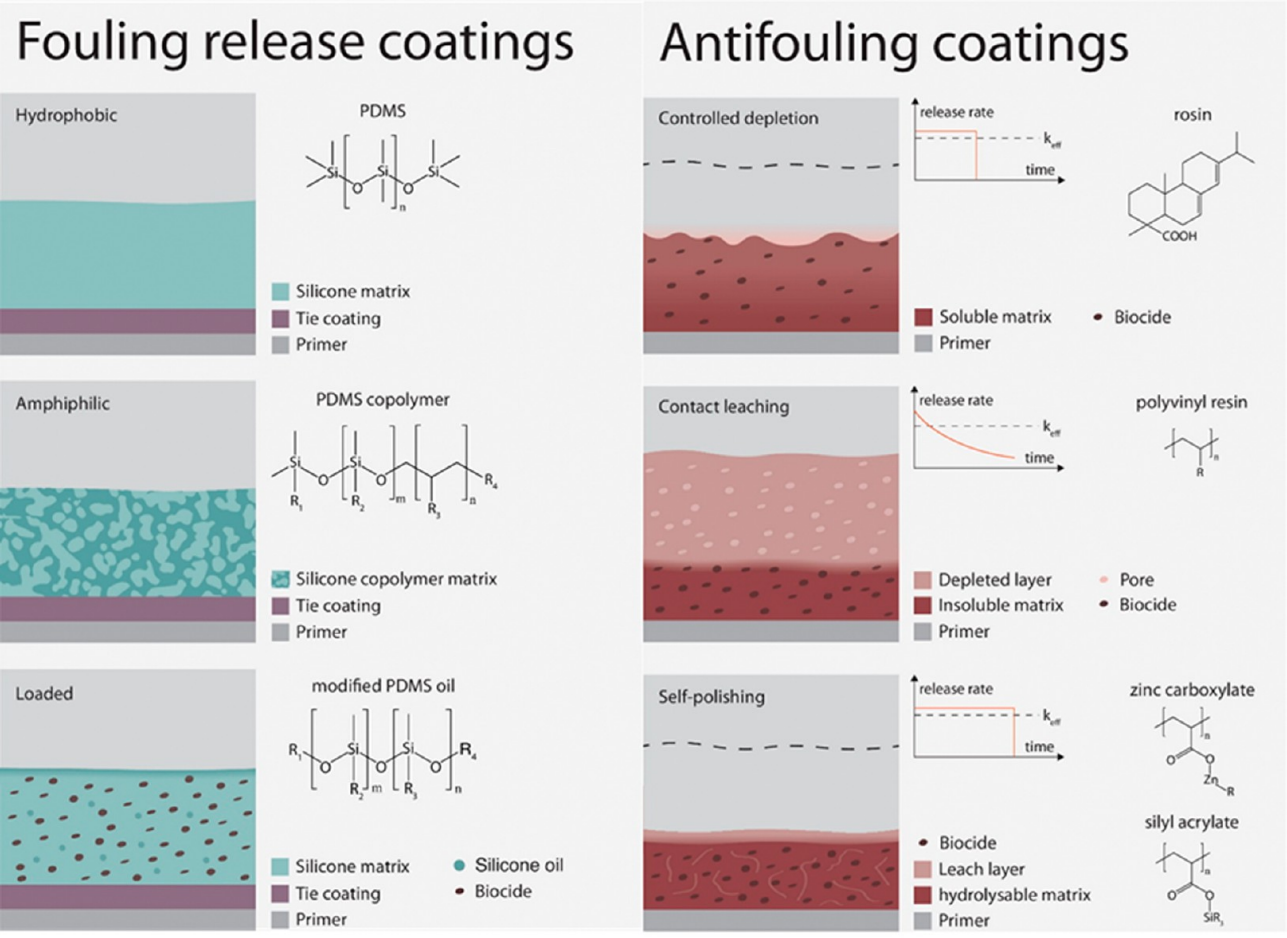
Illustration of the fouling release coating and antifouling coating. Reproduced with permission from ref (94). Copyright 2023 Taylor and Francis.

Coatings have been an excellent strategy to combat biofouling and protect marine sensors. However, coatings must be transparent and environmentally friendly when they are applied to optical sensors. Therefore, a significant number of attempts have been made to create nontoxic transparent coatings that inhibit the adhesion of fouling organisms. Preventing the settlement of proteins, algae, and sea-immersed objects is initiated by Fouling-resistant coatings. Highly hydrated surfaces, where water networks obstruct any foulant attachment, are the subject of this kind of coating. As a result, a tightly packed layer of water forms, which acts as a free energy barrier; subsequently, this layer maintains a distance from the fouling particles. Figure 12 illustrates the different types of fouling release and antifouling coatings for all the marine submerged surface.^94^ Fouling release coatings are ecofriendly, biocide-free coatings. They reduce the adhesion force that exists between the surface of the coating and the fouling organisms.

Antifouling coatings are dominated by biocide antifouling, whose effectiveness depends on controlled biocide release. Generally, the application of grafting polyethylene glycol (PEG) to surfaces to create PEG brushes is a conventional method employed to impede the adsorption of protein molecules onto the submerged surfaces.^95,96^ PEG is a biocompatible material that is water-soluble, nontoxic and highly flexible, which has motivated research and production efforts to create an antifouling coatings system for marine sensors.^97^ Its large hydration layer, quick conformational changes, and steric repulsion have all been implicated in its effective foulant repulsion. Numerous additional organizations have been using a variety of grafting procedures and coating other substrates with PEG of varied designs.

#### Fouling-Release Coatings

In recent decades, a significant number of attempts have been made to create environmentally friendly coating systems to inhibit the adherence of fouling organisms.^98^ The primary objective of this coating is to diminish the adhesion strength of the organisms to the submerged surface.^99^ Previously, we discussed a few polymer systems based on fluoropolymers and siloxane elastomers and their copolymers to combat foulants due to their diminished elastic modules and low surface energy. For instance, hybrid xerogel has been showcased for its antifouling characteristics attributable to its repellent qualities against the settlement of zoospores from the Ulva seaweed species.^100^ Furthermore, given that the surface of the coating exhibits hydrophobic properties, a pure fouling release coating can solely be effectively employed in dynamic environments. Effective fouling-release coatings have a smooth surface, low modulus (to aid detachment), and low surface energy (to prevent mechanical interlocking).^101^ To design foul-release coatings, fluorinated polymers and silicone elastomers are considered as suitable antibiofouling materials. Thus, nowadays, various research groups have been using these hydrophobic polymers, although they possess high chemical stability in contrast to PEG in Fouling-resistant coatings. Furthermore, because of its high crystallinity and limited solubility, PTFE is challenging to treat and bind to surfaces.^4,102^ As a result, other fluorine-containing polymers like perfluoropolyether and fluorinated (meth)acrylates have gained attention instead of fluoropolymers. Krishnan et al. proposed a highly intriguing alternate strategy that included fluorinated comb-shaped liquid crystalline block copolymers.^103^ The polystyrene block served as a compatibilizer and gave the system solubility, while the liquid crystalline phase was found to prevent surface rearrangement.^104^

To combat foulants such as Webster tackled problems, siloxane-polyurethane based antifouling systems specifically hybrid coatings composed of 30 wt % PDMS and the predominant PU component have been utilized as self-healing coatings.^105,106^ Similarly, Galhenge and his colleagues show that lubricating the surface with silicone lubricants produced considerably more improved qualities.^107^ They have reported that only 1 wt % of oil could be highly effective for the reduction in adhesion of macroalgae, barnacles, and mussels to submerged surfaces. Furthermore, PDMS-based coatings present various challenges, including the chemical and processing of coatings. Martinelli et al. presented a novel technique to reduce these issues by using UV light to photo-cross-link methacrylic PDMS oligomers at ambient temperature without the need of hazardous catalysts for use as antifouling coatings.^108^Figure 12 illustrates the different types of fouling release and antifouling coatings for all marine submerged surfaces.

### Biocide Coatings

The main goal of biocide coatings is to stop the sticking process or even kill the unwanted organisms when they try to attach to the treated surface.^109^ Biocide systems can be classified into two types based on how they work: contact-killing surfaces or surfaces that release antibacterial agents. In the first method, chemical groups that kill bacteria are placed on a surface so that they can kill anything that touches it. In the second method, these Chemicals stop bacteria from attaching to the surface before they can stick.^110^ The main use of coatings that prevent fouling is in healthcare. They are designed to stop bacteria from sticking, which can lead to inflammation. Also, since these tiny fouling organisms are important for bio fouling, biocides could be useful in the marine industry too. Furthermore, biocide coatings are also promising for use in the marine industry, as such materials play important roles in the inhibition of biofouling phenomena.^111^ On the other hand, for organic coating, organotin materials are incorporated physically instead of chemically bonded to a polymer matrix. This led to the free removal of biocides from the matrix, which reduces the coating’s service life.^112^ To minimize this issue, various reports have been demonstrated to maintain a steady leaching level of the organotin compounds from the polymer matrix.^112,113^ Likewise, many ancient societies talked about how silver materials can fight germs. Using silver to prevent the building up of attachment is one of the oldest methods known.^114^ It is said that Ag(I) ions are very good at helping to release things, even in very small amounts (less than 1 μg per millimeter). They can also help fight bacteria that do not respond to antibiotics.^115^ In the past ten years, industries have gained a lot of interest in using silver-based surfaces that kill bacteria.^116^

#### Photocatalytic Coating

Photoactive coatings involve the process of degradation of foulants upon irradiation by light. During this process, the photosensitizer materials absorb light and pass energy to oxygen or water creating very harmful reactive oxygen species (ROS).^117^ Nowadays, titanium dioxide-containing materials are the most researched for their ability to work as coatings that can be activated by UV light.^118^ By adding the TiO_2_ particles, Wei et al. enhanced the ability of Cu/epoxy composite resins to break down dirt.^119^ They have reported that the incorporation of 1 wt % TiO_2_ results in a significant enhancement of the antibacterial properties. This can be confirmed with complete eradication of *E. coli* bacteria in 2 h in the presence of sunlight. Similarly, by including graphene oxide^120^ or hiding the nanoparticles with a layer of graphene,^121^ the enhanced photocatalytic property was demonstrated due to inhibition of particle aggregation. Furthermore, TiO_2_ materials have been extensively used for self-cleaning coatings.^122^ This substance can exhibit both superhydrophilic and photocatalytic photoinduced characteristics. Consequently, TiO_2_ surfaces become extremely hydrophilic when exposed to UV light, improving their antifouling qualities.^123^ Similarly, a few studies also demonstrated the same phenomenon for ZnO.^124−126^ It was revealed that nanometer-sized TiO_2_ particles enhance the wettability property and function as photocatalysis when exposed to sunlight.^123^

#### Electrochemical Antifouling Coatings

Electrochemistry is an alternative method that can be used as a practice to design fouling-repellent coatings. Furthermore, an alternative strategy to stop marine sensor fouling organisms is the electrolysis reaction, which produces chlorine and hypochlorous acid. To provide fouling resistance potential, this technique uses an electrode next to sensors or through a conductive layer on the sensor surface. However, susceptibility of the coating to be damaged during the application in seawater is a major concern.^127^ Alternatively, other materials such as graphite-silicon electrodes^128^ and titanium nitride (TiN)^129^ have also been studied for electrochemical processes to combat biofouling. The application of electric pulses for combating biofouling organisms such as hydrozoans has been demonstrated.^130^ Furthermore, Delauney et al. have used a transparent conductive tin dioxide (SnO_2_) coating for biofouling protection for TriOS fluorometers (Ammerland, Germany).^131^ Moreover, a copper layer can be deposited at the edge of the optical window to enable electrical contact with the SnO_2_ coating because this conductive layer functions as an anode, with polarized potential to produce chlorine. The TriOS (Ammerland, Germany) fluorometers were discovered to have been reinstalled with new optical windows using the fully integrated electrochemical array.

## Field Tests for the Application of Antifouling Coatings

The occurrence of biofouling presents a significant obstacle for sensors used in monitoring water quality in the ocean environment. This greatly affects the functioning and data integrity of the sensors. In principle, marine sensors and their housing materials are particularly vulnerable to biofouling since organisms can cling to the submerged parts of the sensors, disrupting their precision increasing weight, and creating a drag on the mooring system, which can lead to deterioration of the structure over time.^132^ The growing need for effective biofouling management prompts various initiatives to create robust and cost-effective techniques for quantifying, analyzing, and evaluating biofouling.

**Figure 13:**
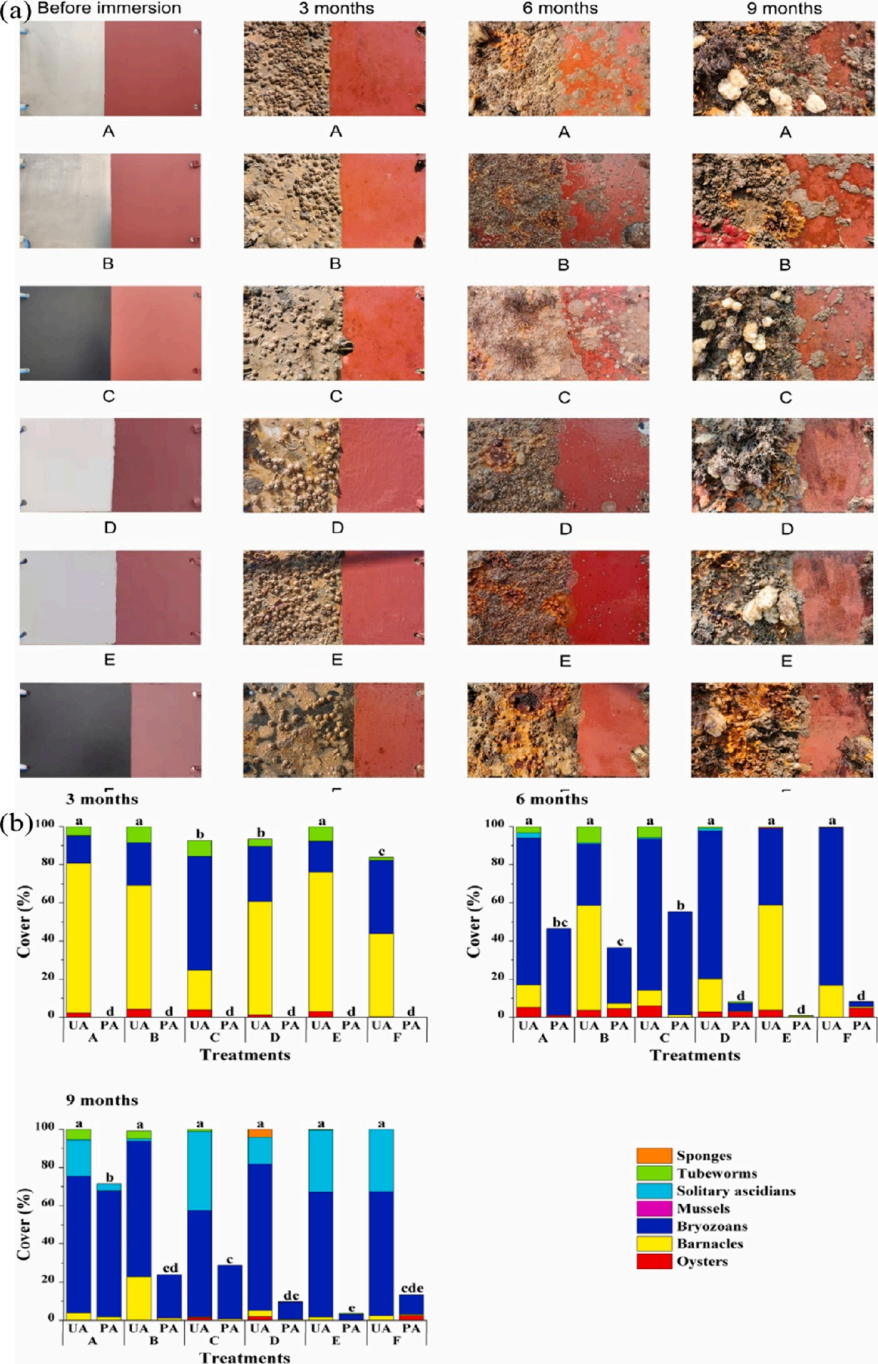
(a) Different types of specimens submerged in seawater for different durations of time; (b) Coverage of fouling organisms on different materials for different times. A: 316 L stainless steel, B: TC4 titanium alloy, C: 7075 aluminum alloy with a black anodic film on the surface, D: polyoxymethylene, E: poly(vinyl chloride), and F: 7075 aluminum alloy coated with Teflon. Reproduced with permission from ref (133). Copyright 2022 Elsevier.

Adrien et al. suggest a categorized system for biofouling development on materials placed in the ocean for extended periods.^132^ They conducted the field trial test for the biofouling colonization analysis at Poolbeg Marina in Dublin materials such as copper, polyoxymethylene, 316 LN stainless steel, and two commercially available antifouling paints (AF paint Micro and AF paint Trilux). Upon submersion in seawater for one year, they observed that commercial antifouling paints were not attached to biofouling organisms. They found that antifouling paint remained free of biofouling (barnacle colonization) after one year of immersion in seawater. However, other materials revealed relatively higher coverage starting from summer to autumn. This observed decline can be attributed to the arrival of winter, the change in water temperature, the number of hours exposed to daylight, and the arrival of winter. Furthermore, it was observed that stainless steel surfaces were exhibited with the highest barnacle colonization at the end of their deployment. However, the commercial antifouling paints showed very low colonization. The rapid deterioration of the biocide matrix that comes into contact with water may be the cause of the antifouling paints declining protective effectiveness over time. However, between the two paintings, barnacle colonization differed significantly. Furthermore, they found that copper shows very little colonization by fouling because it is a toxic material for organisms, which consequently enables it to achieve the highest fouling rating score of nearly 100%. The efficiency of an antifouling paint containing the natural compound camptothecin (CPT) was investigated by Hao et al.^133^ on six distinct materials including 316 L stainless steel, TC4 titanium alloy, 7075 aluminum alloy, polyoxymethylene, polyvinyl chloride, and Teflon. Furthermore, they constructed an underwater sensing housing using these materials. For nine months, the panels were suspended on a floating raft in Xiamen Bay, China (24°52′ N, 118°17′ E) after being painted with prepared CXÅT based paint. Additionally, a spectrophotometer, fluorometer, and visible fluorimeter were used to investigate the antifouling performance of the films via analyzing the COD, BOD, and chlorophyll concentration, respectively. As shown in Figure 13a and b, a tiny percentage of fouling organisms were seen on the unpainted portion of each panel after three months of their submersion in seawater. However, virtually no macrofouling was seen on the painted portion of the panels. On the other hand, CPT-based paint demonstrates outstanding antibiofouling performance. The panels fabricated with polyvinyl chloride, 316L stainless steel, and CT4 titanium alloy displayed 100% microfouling coverage.

**Figure 14:**
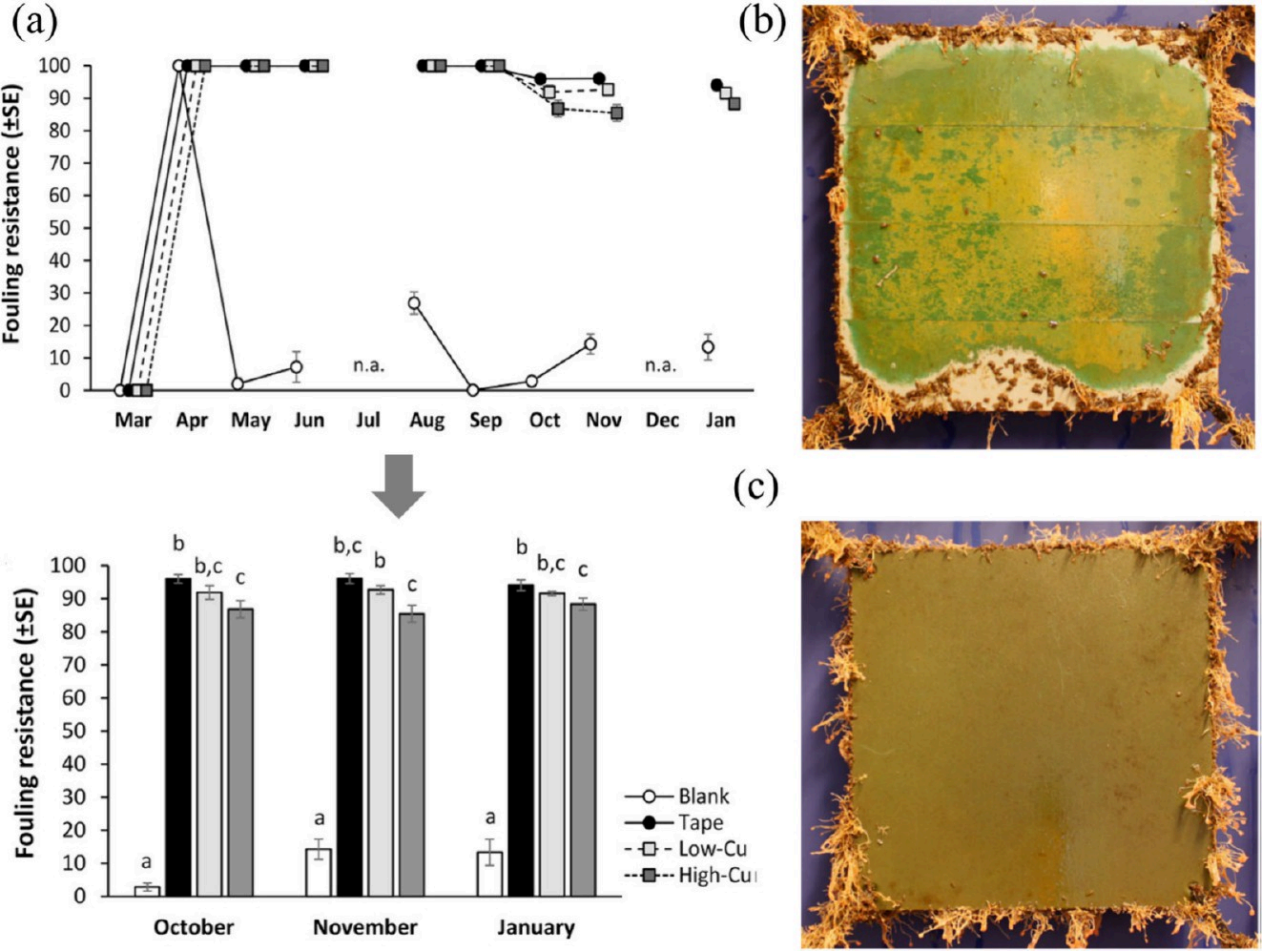
(a) Measurement of antifouling resistance of the sample during the period of March–May and July–November. Lowercase letters above bars indicate the results of pairwise comparison for levels of the factor “Coating” within “Coating × Time”. (b) PVC panel, where copper was leached out; (c) Copper embedded antifouling film. Reproduced with permission from ref (19). Copyright 2021 Elsevier.

After four months of use in a marine environment under a surface buoy, the housing of the underwater spectrophotometer, fluorimeter, and wiper systems painted with CPT remained clean. Additionally, unpainted stainless-steel surfaces that protect the sensors were attached to barnacles. Additionally, after six months of immersion in the same sea area, the painted housing of the UV fluorometer remained clean. Still, the unpainted portions were heavily fouled primarily by bryozoans, barnacles, and tubeworms. Bloecher et al.^33^ assessed the antifouling characteristics of the PU films containing varied concentrations of copper. They used two different antifouling PU films, which were incorporated with a copper concentration of 586 and 306 g m^–2^ by using cold spray technology. Furthermore, they considered two more films, including an untreated adhesive film (blank) and a commercial copper shim tape (copper concentration of 367 g m^–2^) to evaluate the antifouling performance against PU films. As shown in Figure 14a, the blank sample was completely contaminated with fouling organisms, which remained below 30 after 1 month of deployment at sea. After trial, it was observed that the fouling resistance of the low copper-contained film was minimal in January, and the fouling resistance on the high copper-contained film was lowest in November. Furthermore, as shown in Figure 14b, the PVC panel was oxidized in seawater indicating a green color and the gray color on the panels reveals its leaching process. Similarly, Figure 14c demonstrates the absence of any oxidation and leaching process, which confirms the antifouling property of the panel after 10 months of sea trial.

**Figure 15:**
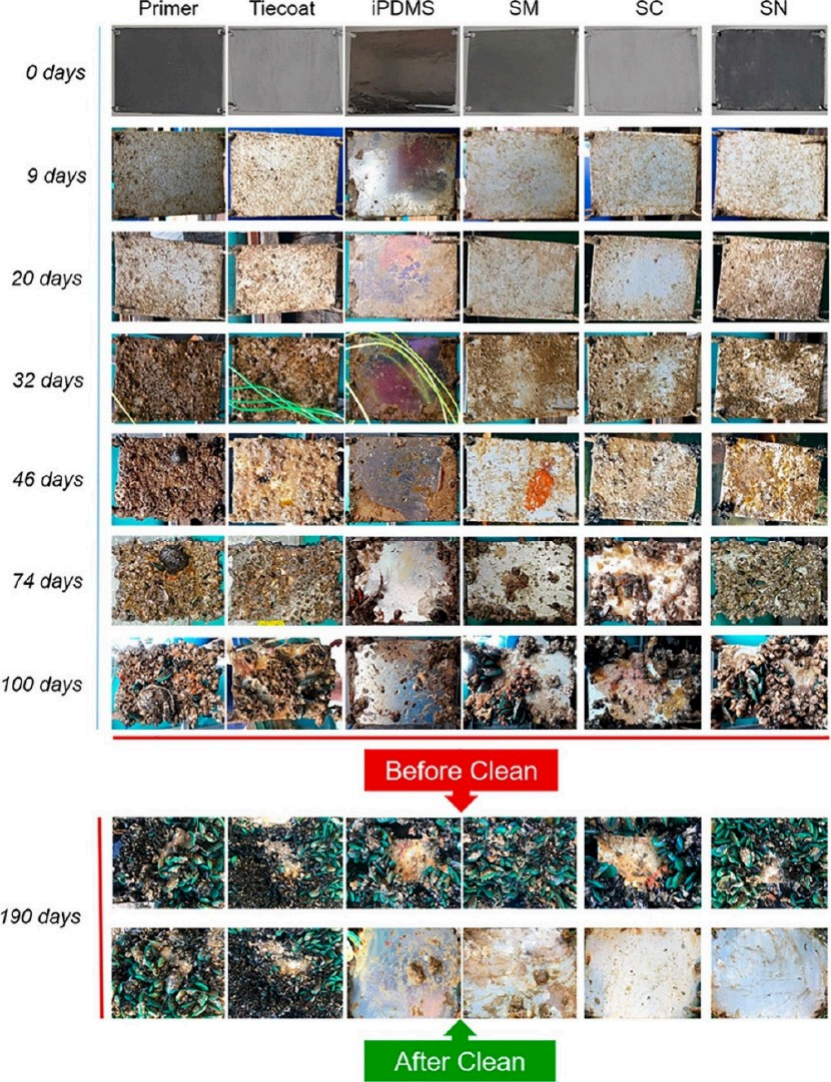
Field trial studies for all seven types of samples were conducted to evaluate the antibiofouling performance. Reproduced with permission from ref (134). Copyright 2020 American Chemical Society.

Inspired by nature, the SLIPS surface is considerably used as an antifouling coating for marine sensors. Moreover, the SLIPS surface has shown exceptional results against fouling organisms. To carry out field trial studies, Basu et al.^134^ investigated the antifouling performance of seven types of samples including three commercial surfaces ((SM47i-02 (SM), SLIPS SeaClear (SC), and SLIPS Foul-Protect N1 (SN)), PDMS, iPDMS (lubricant infused PDMS), one primer coating, and a tie coating. All of the samples were coated on Aluminum plates. They deployed all samples at different locations in Singapore water to conduct field trial studies under two different conditions such as stagnant water and hydrodynamic force. All commercial coatings along with three control coatings (primer, tie coat, and iPDMS) were placed underwater and checked visually at different times for over 6 months. After that panels were cleaned to see how easy or hard it was to remove any build up, as shown in Figure 15. It was revealed that laboratory tests showed no major effects from dirty coatings on the samples. However, when the coatings were placed in still seawater, they worked well to prevent large sea organisms from sticking to them. Moreover, under strong water flow conditions, some weakly attached films were found. These results confirm that these coatings show excellent performance against the resistance to the attachment of marine foulants.

## Impact of Oceanographic Fouling on Marine Sustainability

Antifouling coatings or strategies are applied to protect sea-submerged materials including marine sensors and their housing materials, boats, ship hulls, etc. to avoid the accumulation of fouling organisms and to improve the measurement performance, including the navigation system. Furthermore, the emission of antifouling paints from the submerged surface to the seawater poses a threat to the environment and among others leads to the banning of TBT and maybe others in the future, developing research for new materials and important methods. However, marine sensors are smaller in size compared to those of the ship hull and boat surface. Therefore, in this section, we have reviewed the impact of the antifouling coatings used not only for marine sensor surfaces but also for other sea-submerged surfaces, including ship hulls, boats, etc.

The impact of commercially available antifouling paints, which contain biocide, has been a serious concern for the ocean environment. Copper biofouling systems such as copper coatings or tapes in sensors define the use of copper or copper alloys as a method to prevent or reduce the buildup of marine organisms on sensor equipment. Copper is an effective biocide against a wide range of marine organisms including barnacles, mussels, and algae. When copper connects with seawater, it starts to release copper ions, which are toxic to these organisms and again prevent them from attaching to surfaces. Generally, copper-based biofouling coating systems assist in keeping sensor equipment free from biofouling and maintaining their performance. Some moored instruments designed to measure conductivity, temperature, and dissolved oxygen (CTDs) can have antifoulant devices applied to them to prevent the attachment of marine organisms. It is also important to consider the environmental impacts and the potential for the chemicals to leach out into the surrounding waters.

## Commercial and Industrial Aspects of Marine Coating Industries

To develop effective antibiofouling strategies from an industrial perspective, it is necessary to face the challenges that involve the production of micro/nanostructure antibiofouling coatings, and other biofouling mitigation methods via mechanical strategies such as the use of wipers, brushes, scrappers are complicated to manufacture for industries. These challenges and considerations regarding the development of new antifouling coatings including the marine environment by marine industries are mainly focusing on environmentally friendly solutions. Because these methods are not only likely to develop antifouling eco-friendly coatings but also try to reduce long-term harm to the environment. In principle, most marine industries widely use coating or cleaning systems to mitigate biofouling organisms. It is reported that only the U.S. Navy has spent more than 1 billion USD annually over the past decades, while global expenses are exceeding 15 billion USD annually.^135,136^ However, fouling release coating holds 64% of the market share, which is the largest share portion of marine coatings, followed by anticorrosion and self-cleaning coatings. Among all of these aspects, the two most important aspects that drive the growth of the marine industrial coating market are the transportation of goods by sea and creational sailing. Additionally, leisure boating is also considered to be the second largest driver for the marine coating market. So, all these factors including shipping, leisure boating, transportation, etc. promote the growth of demand for marine coating. It is reported that 36% more electric power is required to maintain the same speed it goes through the water when a hull surface is covered by only 10% barnacles fouling. So, this indicates that more than 110 tons of excess carbon emission additionally cost $6 billion of fuel cost for shipping industries.^94^ Moreover, nowadays marine coating manufacturers are stressing how to improve marine sustainability, including other performance indexes such as power consumption, fuel consumption, and carbon emission.

Nowadays, most marine hull coatings that are available on the market are biocidal-based coatings. In 2011, it was reported that nearly 94% of all marine coatings are considered biocidal coatings.^137^ However, in 2014, the market share was reduced by 90%, which indicated the shifting of industries toward new technologies-based marine coatings.^138^ Here, we have presented the commercial aspect of the antifouling coating fouling release coatings by different manufacturers in Table S2 (Supporting Information).^94^ Although biocidal antifouling coatings are widely used as hull coatings, they release biocidal molecules via the diffusion process. Furthermore, modern antifouling coatings also face challenges such as low biocide coating, short life span, robustness, etc. Due to these challenges, modern antifouling coatings are produced with the combination of stability of high molecular weight polymers and the release of biocidal molecules in a controlled manner using ion exchange process technology. Today, most antifouling coatings markets are Zn/Cu acrylates-based self-cleaning polymer (SPC) products as presented in Table S2 (see Supporting Information). However, beyond academic research, AkzoNobel produced a fouling release coating to boost its performance.

## Challenges and Future Prospects

The process of biofouling is a natural one that can cause malfunctions to the measurements of the sensors within a week. Therefore, it is necessary to develop efficient and environmentally safe antifouling strategies and further carry out measurement studies on how the use of antifouling coatings affects sensor performance in the intended environment. Currently, commercial antifouling coatings used for marine sensors face problems and represent only 1% of the total marine coatings market. However, coatings to combat marine biofouling will likely be based on what is “eco-friendly” or biocides-free. In this review, we have presented significant research efforts that are being supervised toward the development of novel, nontoxic, including physical and chemical strategies combating foulants adhered to marine sensor surfaces. We have illustrated several examples to know the effect of wettability theories and nanomicro scale engineered coating designs on combating biofouling organisms or prevention and settlement of microorganisms on the surfaces. Furthermore, the practical application of antifouling surfaces requires outstanding chemical stability, mechanical and high/cold temperature resistance, UV resistance, and higher transparency and robustness.

Furthermore, the marine industries have a great capability to double their contribution to the global economy by 2030. Since real-time measurements and long-term data collection under the water environment are impacted by the accumulation of marine organisms on the submerged surfaces, However, improved monitoring of the ocean parameters, the underwater environment, resources, and operations need to be based on solutions that do not threaten the ocean’s health. In the future Ocean approach, data are collected through several measurement platforms in the sea, such as coastal areas, ships, boats, and radio-controlled or autonomous vessels. Many research and development challenges are associated with the World Ocean Council approach including the best possible measurement, low energy, and low-cost underwater wireless communications, and software systems to develop sensors for the measurements over larger areas. Among the significant strategies, the development of antibiofouling coating is recognized as a key strategy for optimizing monitoring systems for high stability and measurement quality to ensure reliable data over longer times and larger surfaces as well.

## Summary and Outlook

This Review discusses the current practices, including fabrication techniques, materials, impacts on the economy, and environmental and existing problems in the development of antifouling coatings for ocean sensors. Nowadays, antibiofouling coatings should possess wide pertinency and endurance to complex marine environments for long times with minimal/no growth. Considering all of the requirements altogether, it remains very challenging to fabricate an efficient and durable antifouling coating. Currently, commercial coatings that include biocides are less harmful than TBT coatings, yet they may still affect marine ecosystems. The study aimed at creating eco-friendly and fouling prevention coatings is widespread, and approaches pursuing most modalities are investigated to minimize the biofouling on deployed devices. At present. The primary design concept involves integrating wettability principles, fabrication methods, design, and sensor materials units into a single coating.

Bioinspired superhydrophobic surfaces and irradiation techniques such as UV or ultrasonic methods have inherent antifouling characteristics. Table S3 (Supporting Information) demonstrates the different features of the various coatings. No single chemistry and surface structure involved in designing antifouling coatings has been identified as the universal antifouling strategy. Sensors have become small-sized, technically advanced, and focused on different applications. Overall, antibiofouling is perhaps the most difficult problem to address for marine sensing. With the high research activity in the field, we believe that the research activities will become increasingly mature with the development of materials. Among all the strategies used for combating biofouling phenomena, UV radiation techniques show the most promising results during field trial experiments and can be further redesigned for future implementation. There is still a lot of research needed to create robust and eco-friendly coatings or technologies to prevent biofouling on ocean sensors. In the future, the focus should be on making coatings that work well together using different methods to stop biofouling. However, it is important to consider factors like being good for the environment, being cost-effective, being easy to produce in large amounts, and working in different ocean conditions. Although a single conventional solution is highly unlikely to reduce the biofouling of the oceanographic sensors. We need better strategies to prevent or mitigate fouling completely for marine sensors that are deployed in harsh conditions in ocean environments. To solve this problem, we need to create standards that require both sensor makers and users, like marine biologists and local monitoring groups, to follow certain rules during field tests before permanent use.

## Abbreviations

SLIPs: Slippery liquid infused porous surfaces
PDMS: Polydimethylsiloxane
PEG: Polyethylene glycol
CAH: Contact angle of hysteresis
CB: Cassie–Baxter
OCA: Oil contact angle
WCA: Water contact angle
PTFE: Polyetetrafluoro ethylene
SBS: Styrenebutadiene styrene
PU: Polyerathene
HNT: Halloysite nanotubes
TEOS: Tetraethyle orthesilane
MPTMS: 3-Mercaptopropyl)trimethoxysilane
COP: Cyclo-olefin-polymer
PFOTS: Perfluorodecyl tricholoro silane
MAPOSS: Methacryl polyhedral oligomeric silsesquioxane
FMA: Perfluorooctyl ethyl methacrylate
PFTS: 1*H*,1*H*,2*H*,2*H*-Perfluorooctyl-trichlorosilane
PAA-Br: Polyacrylic acid-bromine
PNIPAm: Poly-*N*-isopropylacrylamide
PSPMA: Poly-3-sulfopropyl methacrylate potassium salt
PVDF: Polyvinylidene fluoride
SRB: Sulfate-reducing bacteria
MMC: Metal matrix composite
CTDs: Conductivity, temperature, and depth
TSS: Total suspended solids
NTU: Nephelometric turbidity unit
TBT: Tributyl tin
HFPB: Hyperbranched fluoropolymer
PVC: Polyvinyl chloride
DMDEOS: Diethoxydimethylsilane
iPDMS: Integrated polydimethylsiloxane
WSN: Wireless sensor network

## Vocabulary

Superhydrophobic surface: Surface that demonstrate the water contact angle more than 150 degrees
CAH: Contact angle difference between the advancing and receding angle of the surface
Superamphiphobic surface: Surface which repels both oil and water is called as superamphiphobic surface
Under water oleophobic surface: Surface which repels oil under water environment is called underwater oleophobic surface
Biocide: Chemical compound that prevents the growth of the microorganism
Biofouling: Accumulation of fouling microorganisms on the surface that immersed in the seawater
