# Supplementary material for: Antibiofouling Coatings For Marine Sensors: Progress and Perspectives on Materials, Methods, Impacts, and Field Trial Studies
Source: ACS Sens. 2025 Mar 5;10(3):1600–19. doi: 10.1021/acssensors.4c02670 (PMC11959602; doi:10.1021/acssensors.4c02670)
Supplement: Supplementary file 1 — se4c02670_si_001.pdf [file se4c02670_si_001.pdf]

# Supporting information

# Antibiofouling Coatings For Marine Sensors: Progress and Perspectives on Materials, Methods, Impacts, and Field Trial Studies

Bichitra Nanda Sahoo[a*], Peter James Thomas[b], Paul Thomas[a], and Martin Møller Greve[a*]

[a]Nanophysics Group, Department of Physics and Technology, Allegaten 55, University of Bergen (UiB), 5007, Bergen, Norway

[b]Measurement of Science Group, Norwegian Research Center (NORCE), Nygårdsgaten 112, 5008, Bergen, Norway

*Email: bichitra.sahoo@uib.no, Phone no: +47 96724065

*Email: martin.greve@uib.no, Phone no: +47 55580000

**Contents**

**Figure S1.** *Schematic showing key concepts for surface wettability*

**Figure S2.** *(a-c) Images of a superhydrophobic lotus leaves (Nelumbo nucifera) with self-cleaning properties at different magnifications, (d-f) SEM images of the lotus leaf illustrate the hierarchical structures. Reproduced from Ref. [10]. Copyright 2009 Elsevier, (g) A photograph of antifogging mosquito eyes. Even though they are exposed to moisture, the surface of the eyes remains dry and clear while the surrounding hairs nucleate many drops, h) An SEM image of a single mosquito eye. i) An hcp microhemisphere (ommatidia). j) Two neighboring ommatidia. k) Hexagonally nanonipples covering an ommatidial surface.*

**Table S1.** Materials used in sensor housing body, connections, and sensor head.

**Table S2.** *Overview of the main commercial coatings systems of the leading manufacturers available in 2023.*

**Table S3.** *Summary of biofouling control for sensors listing working principles, materials and technologies performance, and strengths and weakness*

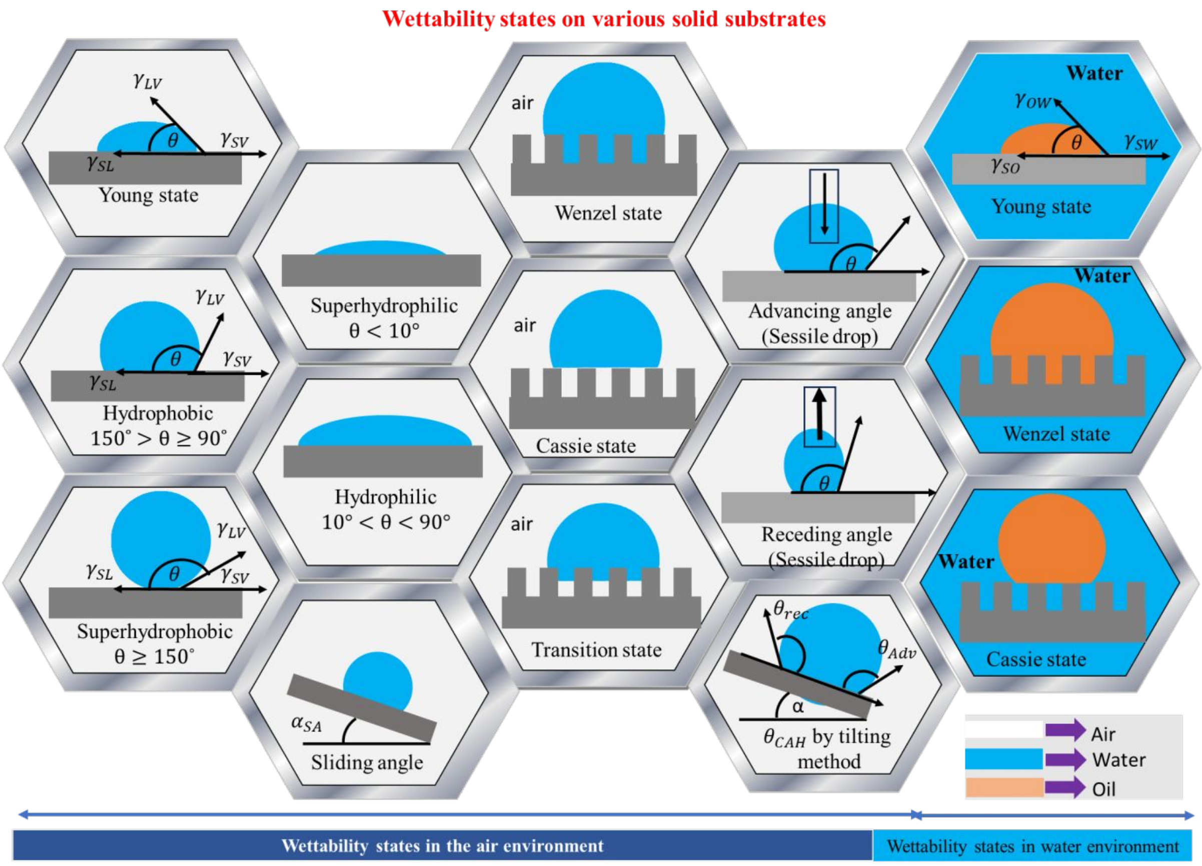


**Figure S1.** *Schematic showing key concepts for surface wettability*

**Wettability theories.** Based on equation shown in Equation 1 [1] and In Figure S1, when a sessile water droplet lands on a surface, a measurable contact angle 'θ' of the sphere-like droplet between the three-phase system, solid/liquid/vapor, can be determined to study the wetting behavior of the surface. This is defined as Young's contact angle, which is valid only for flat surfaces. [2]

$$Cos\theta = \frac{\gamma_{sl} - \gamma_{sg}}{\gamma_{lg}} \quad (1)$$

where '$\gamma_{sl}$' is the interfacial energy between surface and liquid, '$\gamma_{sg}$' is the interfacial energy between the solid and air, and '$\gamma_{lg}$' is the interfacial energy between liquid and air environment. The measurement of droplet mobility or sliding angle ($\alpha_{SA}$), further illustrates the repellency of the surface. Furthermore, another important wetting parameter is the contact angle of hysteresis (CAH) '$\theta_{CAH}$' which is the difference between the advancing and receding angles of the droplet on the surface. This decides the self-cleaning property of the surface. This can be measured by the sessile drop and tilting method as shown in Figure S1. Additionally, topography features on surfaces also play a critical role in the wettability of the coatings. The

effect of topographic features on the wettability property of the surface, are described in terms of Wenzel and Cassie-Baxter equations 2 and 3 respectively [3, 4].

$$Cos\theta_W = r\frac{\gamma_{sl}-\gamma_{sg}}{\gamma_{lg}} \quad (2)$$

$$Cos\theta_{CB} = \frac{\gamma_{sl}-\gamma_{sg}}{\gamma_{lg}}rf + f - 1 \quad (3)$$

As shown in equation 2, '$r$' is the roughness of the surface and can be defined as the ratio of the area fraction of the rough surface divided by the projected flat surface, '$f$' is air fraction, $'\theta_W$' and '$\theta_{CBW}'$ are the Wenzel's and Cassie-Baxter's contact angle on the heterogeneous surface. For the Cassie-Baxter equation, the contact angle of the surface is determined by the heterogeneous surface with cavities below the droplet. For a moving droplet, the maximum and minimum contact angles are called advancing and receding contact angles, respectively as shown in Figure S1.

Besides the liquid wettability in an air environment on solid substrates, the oil wettability phenomena in the water environment is also described in Figure 2. The oil droplets on flat or smooth surfaces contribute to the oil/water/solid three-phase interface. The three-phase apparent OCA under this condition demonstrates the following 4 [5].

$$Cos\theta_{OW} = \frac{\gamma_{sw}-\gamma_{so}}{\gamma_{ow}} \quad (4)$$

In equation 4, $\gamma_{sw}, \gamma_{so},\ and\ \gamma_{ow}$ refer to the interfacial tension of solid-water, solid-oil, and oil-water interfaces, respectively. Taken into consideration that the aforementioned in-air Young wettability can be effective for both water [2] and oil droplets [6] on ideal flat surfaces, the relevant WCA ($\theta_W$) and OCA ($\theta_O$) are given using the in-air Young's equation of water (Equation 5) and oil (Equation 6), respectively.

$$Cos\theta_W = \frac{\gamma_{SA}-\gamma_{SW}}{\gamma_{WA}} \quad (5)$$

$$Cos\theta_O = \frac{\gamma_{SA}-\gamma_{SO}}{\gamma_{OA}} \quad (6)$$

Here, $\gamma_{so}, \gamma_{OA},\ and\ \gamma_{WA}$ refer to the interfacial tension of solid-oil, oil-air, and water-air interfaces, respectively.

Moreover, surface energy is another important parameter, which strongly decides the interaction and intermolecular forces between the surfaces. Thus, surface energy that strongly influences the formation of biofilm on solid surfaces is studied through Baier curve [7]. It was observed that the solid surface can reduce the adhesion of fouling organisms with a low surface energy of about 22-24mN/m, which results in reduced bio adhesion. However, a surface energy of 50-60 mN/m enhances the interaction between solid surface and fouling organisms.

Similarly, a surface energy of 72 mN/m demonstrates excellent resistance against the adhesion of bio-organisms to the surface, as it induces an active hydration layer, which inhibits the formation of bio-organisms [8,9].

**Natural bio-inspired Surfaces.** To design functional coatings like antifouling coatings, researchers have obtained inspiration from natural biological systems. A classic example is the Lotus leaf which can remove dust particles easily from its surface when water is applied (see Figure S2a-c). This is possible due to the hierarchical structures of the low surface energy crystal on the lotus leaf surface[10] as shown in Figure S2d. Moreover, both Barthlott and Neihuis have revealed that wax crystalloids and stable air cushions in hierarchical structures (Figure S2e&f) on the lotus leaf surfaces play a key role in demonstrating the self-cleaning property of the lotus leaf [11]. By inspiration from these natural features of the lotus leaf, many artificial superhydrophobic surfaces have been produced and applied as antifouling surfaces in modern industries. Similarly, a photograph of Mosquito's eyes [12] is illustrated in Figure S2g, which is comprised of well-patterned hundreds of miniature hemispheres, with nanostructures distributed around each hemisphere. An SEM image of a single mosquito eye is shown in Figure S2h. The eye demonstrates a liquid repellency property due to the presence of microhemispheres as shown in Figure S2i. Furthermore, an SEM image of hexagonally nano nipples covering each microhemisphere is shown in Figure S2j&k. The design of this structure has also inspired new strategies for the preparation of the antifogging and antifouling surfaces[12]. Like Lotus leaf and mosquito eyes, butterflies demonstrate natural examples of antifouling coatings[13]. Microgrooves are arranged side by side on the scales of different types of butterflies' wings and demonstrate anisotropic superhydrophobic properties. Furthermore, it also reveals high adhesion properties, while the droplet rolls off from outside to the inside and anisotropic nature, while water rolls off from inside to the outside of the wings of the butterfly wings. These excellent properties of the butterfly's wings deliver new ideas in the fabrication of directional liquid-repellent surfaces. Similarly, Fish scale structures are a good example of the development of the underwater superoleophobic [14]. Even inside water contaminated with oil, fish scale structures keep themselves clean and demonstrate excellent stain and oil resistance and self-cleaning properties. This is due to the chemical composition and microstructures of the surface.

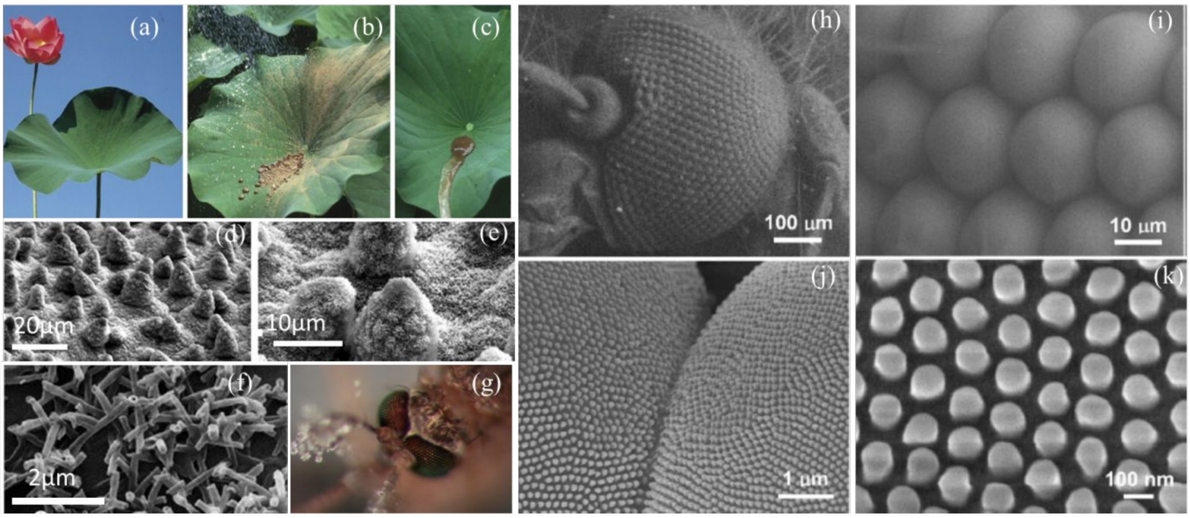


**Figure** S2. *(a-c) Images of a superhydrophobic lotus leaves (Nelumbo nucifera) with self-cleaning properties at different magnifications, (d-f) SEM images of the lotus leaf illustrate the hierarchical structures. Reproduced from Ref.* [10]*. Copyright 2009 Elsevier, (g) A photograph of antifogging mosquito eyes. Even though they are exposed to moisture, the surface of the eyes remains dry and clear while the surrounding hairs nucleate many drops, h) An SEM image of a single mosquito eye. i) An hcp microhemisphere (ommatidia). j) Two neighboring ommatidia. k) Hexagonally nanonipples covering an ommatidial surface. Reproduced from ref.* [12] *copy right year 2007 John Wiley and Sons.*

Fish scales are comprised of calcium carbonate, protein, and a layer of mucus layer, which play a key role in facilitating super oleophobic properties in water and super oleophilic properties in air. Additionally, it exhibits low adhesion to oil droplets and slips off the surface easily [15]. This is caused by the presence of many micro-size papillae with mucus layers, which are radially arranged on the skin. The combination of this micro papillae and mucus layer provides excellent antifouling properties. This not only exhibits antifouling properties but also shows great stain resistance in harsh conditions. In contrast, the lotus leaf exhibits air-filled micro/nanostructures to repel water or low surface tension liquids, the Pitcher plant traps water, which is super hydrophilic with slippery properties. The structure of the pitcher plant is super hydrophilic which is covered with a wax layer that is called hydrophilic nectar. Therefore, water droplets quickly fill the pores to form a homogeneous liquid film on top, making the peristome surface slippery. Based on the nature of the pitcher plant, this is known as SLIP (slippery liquid infused porous) surface. SLIP consists of micro/nanostructured surfaces that is capable of locking lubricating liquid and repels a wide variety of immiscible liquids and solids,

thus providing antifouling and self-cleaning properties. However, the key advantage of SLIP coatings over other coating technologies is their ability to restore the slippery properties after damage, as the repellant liquid will be able to refill the voids [16, 17]. So, this way pitcher plants are inspired to develop artificial coatings that repel various liquids and display the antifouling and self-cleaning properties. However, long-term stability for SLIP surfaces is hindered by damage to the top layer through evaporation and physical shear. Instead of using fragile textured surfaces, thick bulk-porous coating could also lower the sensitivity to abrasion.

**Artificial super-hydrophobic coatings.** In the previous section, we briefly discussed the importance of wettability and topographic features with a few examples of nature-inspired species for the determination of superhydrophobic and antifouling characteristics of the coatings. Critical surface parameters including surface wettability and topographic features are required to be embraced in the design of new marine coatings for biofouling control [18]. In recent years, superhydrophobic surfaces with contact angles of more than 150 degrees have been demonstrated in various research areas, where applications of self-cleaning are of interest[19,20,21]. Thus, it has been confirmed that an efficient antifouling coating possesses low surface energy, and high fouling release properties.

In this section, we briefly discuss two kinds of polymer coatings with fouling release properties such as fluoropolymers, and siloxane. Fluorine-based polymers, for example, PTFE have been considered as an excellent base material for the fabrication of antifouling coatings due to their outstanding chemical stability in harsh conditions, and low surface energy. Therefore, the interest in fluoropolymers shifted to other fluorine-containing polymers such as fluorinated methacrylate and perfluoropolyether. Similarly, PDMS is also a silicone-based hydrophobic polymer material that belongs to the elastomer and has low surface free energy. Due to its hydrophobicity and low elastic modulus properties, it demonstrates excellent fouling release materials performance. However, due to the lack of adhesion to the substrate and low resistance to mechanical damage, the application of PDMS is limited in the marine sector. Therefore, extensive research has been carried out for the fabrication of PDMS-based composites with the incorporation of additives and functionalization to achieve the most efficient biofouling control performance for long-term marine applications.

**Artificial super-hydrophilic.** As discussed in Section 1.2, the high wettability of such surfaces results from surface chemistry, i.e., high surface free energy and surface morphology. Hydrophilic surfaces have always a strong affinity to water and exhibit water contact angles below 90° [22]. Thus, superhydrophilic surfaces with a water contact angle lower than 10° can

be achieved by a proper combination of surface chemistry and roughness features [23, 24]. (Super-) hydrophilic materials have been extensively investigated as antifouling coatings for a wide range of applications in biomedical engineering and the marine industry [25–28]. It was found that the antifouling properties of these materials are caused by the strong surface hydration layer, which acts as a physical and energy barrier for the organism's settlement. Moreover, biological molecules such as proteins, as well as bacteria and other marine organisms, are unable to displace tightly bound surface water molecules, blocking them from attaching to the surface [29,30]Among all developed super hydrophilic coatings, PEG is one of the important hydrophilic polymers that have been concerned in biomedical and marine environments [31,32]. The antifouling properties of this coating are mainly facilitated by the strong PEG-water interfacial energy and the hydration layer forming due to the formation of hydrogen bonding between the water molecules and the ether oxygen atoms [33–35]. Furthermore, due to the electrical neutrality of PEG, the electrostatic interaction with charge protein domains can be minimized, which also prevents protein adsorption by steric hindrance[36–38]. Similarly, several efforts have been made to develop PEG-based hydrogel coatings [39–42].

**Artificial SLIP coatings.** SLIPS demonstrate both low CAH and hydrophobic properties. It was demonstrated that to achieve a good fouling-release performance, both a large water contact angle and a low contact angle hysteresis are required [43]. Thus, a common approach to lower contact angle hysteresis and attain slippery surfaces is to mix oily lubricants into hydrophobic coatings [44,45]. Wong *et al.* were the first to demonstrate the concept of SLIP surface, which was inspired by the pitcher plant. They used micro/nanostructured low-surface energy substrates to lock the infused lubricating fluid via capillary forces [46]. As a result, the surface exhibited liquid repellency, low contact angle hysteresis, and self-healing from physical damage, which confirmed their marine antifouling properties. Similarly, Ba et al. demonstrated the adherence of diatoms to a PDMS surface lubricated by silicone oil [47]. PDMS coatings incorporated with higher viscosity silicone oil exhibited the lowest adhesion and highest removal rate of benthic diatoms. The performance improvement was confirmed by increased hydrophobicity, decreased elastic modulus, and prolonged time of silicone oil leaching. Furthermore, Amini *et al.* constructed a SLIP surface by using an oil-infused PDMS surface to inhibit the mussle's attachment to the surface [48]. It was found that the infused lubricant oil deludes the mussel's mechanosensing ability and minimizes thermomolecular work of adhesion, which results in low attachment frequency and much lower attachment strength. Hence, SLIP surfaces are considered to resist the adhesion of any microorganisms, and the lubricant layer also forms a physical barrier to the corrosive medium and, as a result, enhances

the corrosion resistance of the coating to some extent. Furthermore, Galhenage *et al.* have demonstrated the mechanical durability of a SLIPs surface. They investigated the foul release performance of the Siloxane-PU system with infused silicone oil SLIPS. It was found that the resulting coating revealed excellent mechanical and adhesion to substrate properties [49]. Similarly, Zhang et al. made a self-healing organogel by infusing silicone oil into the PDMS-PU matrix system[50]. It was found that organogel exhibited low sliding and excellent performance in inhibiting bacterial attachment.

The presence of a lubricant layer on SLIPS surfaces only resists the migration of biofouling organisms to the surface. Furthermore, the longevity of the SLIPS surface is very limited due to the leaching of the infused lubricant liquid which can cause loss of antifouling performance. Many studies have demonstrated the robustness of SLIPS surfaces. Similarly, He *et al.* fabricated a nanotextured surface by infusing it with perfluoropolyether to minimize the biofouling problem for optical instruments [51]. Recently, Tenjimbayashi *et al.* designed a lubricant-inserted sandwich-like coating, where a microporous layer was placed on the top of the SLIPS surface to block the loss of lubricant. The coating resisted for at least 45 days in seawater which was much longer than the traditional SLIPS[52]. Although the SLIPS surface has been recognized as a solution for biofouling problems, there is a concern about the impact of the released oil on marine ecology. Thus, the impacts of these lubricants on the ecological system in the long run are still under investigation. Due to the concerns around the environmental safety and high carbon footprint of the production of synthetic oils, plant oils such as argon oil, castor oil or coconut oils have been considered as suitable replacements.

**Artificial superamphiphobic, and underwater super-oleophobic surfaces.** In addition to superhydrophobic surfaces and SLIP surfaces, several studies related to superamphiphobic coating (both water and oil-repellent) for the application of antifouling surfaces have been demonstrated. Earlier, we have discussed the role of superhydrophobic surfaces comprised of micro/nanostructures and surface-free energy in mitigating biofouling problems. However, these surfaces lose their repellent properties, when in contact with various low-surface energy liquids, such as oils including hexane, decane, tetradecane, hexadecane, etc. and other liquids for example, milk, cola, fruit juice, ink, etc.

While various antifouling properties strategies have been implemented, a few strategies demonstrate both self-healing and superamphiphobic properties. For example, Chen et al.[53] fabricated a self-repairing underwater superoleophobic and anti-biofouling coating through a self-assembly of hydrophilic polymeric chain-modified hierarchical microgel spheres.

Moreover, their demonstrated coating can regain the oil and biofouling-resistant properties once its surface is mechanically damaged. Importantly, superamphiphobic materials have been considered as the most attractive materials due to their wide range of applications in many fields, however the fabrication of most superamphiohobic coating is time-consuming and complicated. Similarly, Ou *et al.* [54] also fabricated superamphiphobic coatings that demonstrate self-cleaning antifouling applications. They fabricated chitin nanocrystals with needle-like structures and later fluorine treatment was done to reduce surface energy. These coatings demonstrate good repellence with a high contact angle for low and high-surface tension liquids such as water, decane, and ethylene glycol. Similarly, a reentrant texture, where a concave topographic curvature occurs from the top to the bottom of the structure, has been demonstrated in various shapes such as from mushroom, overhang, trapezoid, undercut, spatula, and sphere to sharp edge [55]. Benefiting from these design structures such reentrant decorated surfaces have received a huge amount of interest, from academic research to the industrial worlds, in diverse applications including self-cleaning, anti-fouling etc. Thus, with an understanding of the balancing roles of surface-free energy and roughness, Tuteja [56] and his coworkers fabricated a re-entrant surface curvature, that can be used to design surfaces, which demonstrates extreme resistance to several low surface tension liquids including decane, hexadecane, octane, etc. Reentrant surfaces

A few authors have been inspired to fabricate oil-repellent surfaces for the application of antifouling coatings in the marine industry of the unique nanostructure surfaces of filefish scale. For example, Huang et al [57] constructed hierarchical films that comprised polypentaflurophenyl arylate free-standing micro pillars grafted with polymethacrylic acid. The obtained films exhibit excellent underwater superoleophobicity and reversible switching between low and high oil adhesion due to the adjustable oil sliding angle. Kobayashi and co-authors[58] have reported the antifouling property for both hydrophilic and hydrophobic polymer brushes bearing nonionic and ionic functional groups prepared by the surface-initiated atom transfer radical polymerization method. This study was designed based on the measurement of oil contact angles of water in air and captive air bubbles as well as hexadecane and silicone oil droplets in water. Transparent structured surfaces inspired by the moth-eye were shown to have anti-biofouling properties. The antibiofouling property is attributed to the surface's topography. Nanoholes on the surface enhance both hydrophilicity and surface roughness. This nanohole creates a surface that inhibits the attachment of microgels due to their smaller size compared to the microorganisms. It is reported that further fluorination of nanostructured surfaces enhances the antifouling properties by reducing the surface energy and establishing a Cassie-

Baxter state [59]. A novel design based on low aspect ratio “pancakes” on sapphire substrates was shown to have self-cleaning properties when immersed in oil-in-water mixtures. The investigation involved contact angle measurements and laboratory exposure to different fouling mixtures. It was also reported that an offshore installation incorporating the windows remained useable for over a year, whereas the normal operation time for standard windows was just 7 days [60].

**Table S1** Materials used in sensor housing body, connections, and sensor head.

| Materials | Applications in a sensor |
|---|---|
| ***Metal and alloys*** | |
| **Copper** | Antifouling material |
| **Titanium** | Sensor housing materials |
| **SS304** | Sensor housing materials |
| **SS316** | Filtration materials |
| **Anodized aluminum** | Sensor housing materials |
| **Titanium** | Sensor housing materials |
| **Nickel** | Sensor housing materials |
| ***Polymeric materials*** | |
| **Polyurethane** | Cable, antifouling materials |
| **Cholroprene rubber** | Cables |
| **Crosslinked polyethylene** | Cables |
| **Poly vinyl chloride** | Cables |
| **HD polyethylene** | Cables |
| **Acrylonitrile butadience styrene** | Sensor housing |
| **FEP Teflon** | Membranes |
| **Polyphenylene sulphide** | Sensor housing |
| **Polyoxymethylene** | Sensor housing |
| **PVDF membrane** | Filtration membranes |
| **Epoxy resins** | Sensor housing materials |
| ***Other materials*** | |
| **Glass** | Optical windows |
| **Fused silica** | Optical windows |
| **Sapphire** | Optical windows |
| **Silicon** | Diaphragms |

**Table S2.** *Overview of the main commercial coatings systems of the leading manufacturers available in 2023. Reproduced from ref.* [61]*. Copyright year 2023 Taylor and Francis.*

| Manufacturer | Name | Technology |
|---|---|---|
| AkzoNobel | Intersmooth | Cu/Silyl acrylate SPC |
| | Interspeed | CDP |
| | Interswift | Blend of Intersmooth and Interspeed |
| Chugoku | Seaflo Neo | CF Z and CF Premium: Cu-free Zn acrylate SPC<br>SL M and SL Z: Silyl methacrylate SPC |
| | Sea Grandprix | 500: Zn acrylate SPC,<br>1000L: Silyl acrylate SPC |
| | Sea Premier | 1000: Zn acrylate SPC<br>3000: Silyl methacrylate SPC |
| Hempel | Atlantic+ | Acrylic SPC |
| | Dynamic | Silyl acrylate SPC |
| | Globic | Nano acrylate SPC |
| | Olympic+ | Acrylic SPC |
| | Oceanic+ | Zn carboxylate SPC |
| Jotun | SeaForce | Ion exchange SPC |
| | SeaMate | Silyl acrylate SPC |
| | Sea Quantum | Silyl metha acrylate SPC |
| Nippon | A-LF Sea | 100:Zn Acrylate SPC<br>250, 400, and 600: Cu-silyl-acrylate SPC |
| | Aquaterras | Biocide free amphilic micro domain SPC |
| | Fastar | Amphiphilic nano domain silyl acrylate SPC |
| | Ecoflex | Original: Cu acrylate SPC<br>Hyb: Cu-silyl-acrylate SPC |
| PPG | ABC | SPC |
| | Amercoat | CDP |
| | Sigma Alphagen | SPC |
| | Sigma Ecofleet | SPC |
| | Sigma Nexeon | Cu-free Zn acrylate SPC |
| | Sigma Sailadvance | Zn methacrylate CSP |
| Fouling release (FR) coatings<br>AkzoNobel | Intersleek | IBiocide-free fluoropolymer |
| AST Inc | SLIPSVR DolphinTM | Biocide-free |
| Chugoku | Bioclean (Biocleanþ) | Biocide-free (Biocidal) |
| Hempel | Hempaguard | Biocidal silicone hydrogel (Actiguard) |
| | Hempasil | Biocide-free silicone enhanced hydrogel |
| Jotun | SeaQuest Biocide-free | Biocide-free |
| | SeaQuest Endura | Biocidal |
| PPG | Sigmaglide | Biocide free pure PDMS |

SPC: self-polishing copolymer; CDP: controlled depletion polymer; CSP: controlled surface-active polymer

**Table S3.** *Summary of biofouling control for sensors listing working principles, materials and technologies performance, and strengths and weakness*

| **Fabrication approaches** | | **Working principles, materials, and technologies** | | **Strength** | **weakness** |
|---|---|---|---|---|---|
| | | **surface energy** | **materials** | | |
| Coating based on wettability | superhydrophobic | • Low surface energy<br>• micro/nanostructures like micro-nano pillar, entrant-reentrant structures etc. | • PDMS<br>• Silicone rubber<br>• PTFE<br>• Fluoro polymer<br>• Carbon black | • Excellent for fouling release materials,<br>• robustness<br>• excellent corrosion property | • Problems with substrate adhesion<br>• Poor mechanical properties<br>• Poor fouling release under real marine conditions<br>• Expensive |
| | Superhydrophilic | • Surface energy<br>• Micro/nano structures<br>• Hydration layer<br>• Polar ionic group | • PEG<br>• Arylate<br>• Hydrogels<br>• Polymer brushes<br>• Epoxy<br>• PEGDA | • Excellent antifouling coating<br>• Low fouling organism adhesion | • Poor mechanical and chemical stability<br>• Poor adhesion<br>• Poor large-scale fabrication |
| | SLIP surface | Low surface energy | • Silicone oil | • Excellent fouling release approach<br>• Low surface wettability<br>• Poor organism adhesion | • Lower substrate adhesion<br>• Lower mechanical strength of the coating<br>• Suitable low scale application |
| | Superoleophobic and underwater superoleophobic | • Low surface energy<br>• Micro-nano structures<br>• Thiol-ene reaction | • Silica fumed particles<br>• Perfluorooctyl trio ethoxy silane (PFOTS)<br>• 8-Methacryl polyhedral oligomeric silsesquioxane (8-MAPOSS)<br>• (1H,1H,2H,2H-perfluorodecyltrichloro silane<br>• TEOS<br>• MPTES<br>• 1H,1H,2H,2H-hepta deca fluoro decyl methacrylate (FMA) | • Excellent for fouling release materials,<br>• Robustness<br>• Excellent corrosion property | • Challenges with mechanical robustness<br>• Problems with Large-scale applications, |
| | Biomimetic coating | • Micro nano structures<br>• Superhydrophobic surface<br>• Underwater superoleophobic surface<br>• Lithography (UV, electron beam | • Lotus leaf<br>• Sharkskin<br>• Rice leaf<br>• Butterfly wing<br>• Fish scale<br>• Mosquito eyes<br>• Pitcher plant | • Excellent antifouling property under laboratory conditions<br>• Low surface energy<br>• High mechanical shear force | • Not suitable large-scale applications<br>• Highly cost<br>• Complexity with fabrication methods |

| | | | | |
|---|---|---|---|---|
| | | techniques, and etching | | |
| **Antifouling strategies** | | **Principles** | **strength** | **weakness** |
| Physical strategies | Wiper technologies | Removal of foulants using brushes and wipers | Easy process for large-scale components or devices | • Not feasible for sensitive components of sensors<br>• higher consumption of power |
| | Using open system | Directly exposed to an environment and promoted for the biofouling process | No complexity and reliability technical problems in the design | • Easily accessible for fouling organisms<br>• Main disadvantage in the difficulty of removing the paint for subsequent redeployments and calibrations and its relative ineffectiveness in keeping the windows clear of fouling |
| | Using closed system | Based on the dissolving biocide technique | • Flushing process should be involved to remove the diluted biocide before performing the measurement<br>• The main advantage is that the sample surface is not required to be exposed to light environment and thus inhibiting the photosynthesis process | • Can disturb the quality of measurement datats<br>• Seas trial has not shown good results so far.<br>• Limited protection of the sensor surface |
| Irradiation techniques | UV irradiation | The process is explored based on the effects of the wavelength of the UV spectrum (100-400 nm) on the DNA of organisms | Uses for filtration membrane, marine sensors, wastewater treatment | • Application to the sensor surface is very limited and in progress<br>• Consumption of energy |
| | Laser irradiation | Prevention of biofouling by barnacle and diatoms | Does't generate any biocide | Requires more consumption of energy |
| | Ultrasonic radiation | This method is based on cavitation phenomena. Cavitation creates a high liquid shear force that disturbs the fouling settlement | Low-frequency sound and vibration are required to unsettle the microorganisms, suitable for large surfaces | Practically not feasible due to the battery operative process and expensive process |
| Chemical strategies | Fouling resistance coating | Inhibiting the settlement of foulants on marine sensor surfaces | Strongly hydrated surface | Poor mechanical strength, weak adhesion of foulants to the sensor surfaces |
| | Fouling release coating | Prevention of the attachment of foulants on solid surfaces | Strongly dependent on hydrophobic, amphiphilic surfaces | • Week adhesion or interaction with the surfaces. |

| | | | | |
|---|---|---|---|---|
| | | | | • Foulants can be easily removed with strong shear or mechanical forces |
| | Biocide coating | Disruption of adhesive mechanism or even killing the fouling species on the surface | Strongly killing microbial organisms | Harmful to marine species and the ocean environment |
| | Photocatalytic process | Using photocatalytic materials, which inhibits the growth of microorganisms | applicable for optical sensors to protect them from marine fouling | The seawater can easily damage the surface. weak adhesion with sensor surfaces. |
| | Electrochemical antifouling method | Based on electrochemistry phenomena. Generation of chlorine and hypochlorous acid by electrolysis of water | Formation of conducting layer on the sensor surface | • Coating can be physically degraded while submerging in water<br>• Consumption of power |

## References


1. (Bonn, D.; Eggers, J.; Indekeu, J.; Meunier, J. Wetting and Spreading. *Rev Mod Phys* **2009**, *81* (2), 739–805. https://doi.org/10.1103/RevModPhys.81.739.
2. Young T. No Title. *Philosophical Transactions of the Royal Society OF LONDON (1776-1886)* **1805**, *95*, 65–87. https://doi.org/10.1098/rstl.1805.0005.
3. Wenzel, R. N. To the Editor : To the Editor : *J. Phys. Chem* **1949**, *53* (9), 1466–1467. https://doi.org/10.1021/j150474a015.
4. Cassie, A. B. D.; Baxter, S. Wettability of Porous Surfaces. *Transactions of the Faraday Society* **1944**, *40* (5), 546–551. https://doi.org/10.1039/tf9444000546.
5. Liu, H.; Zhang, L.; Huang, J.; Mao, J.; Chen, Z.; Mao, Q.; Ge, M.; Lai, Y. Smart Surfaces with Reversibly Switchable Wettability: Concepts, Synthesis and Applications. *Adv Colloid Interface Sci* **2022**, *300* (December 2021), 102584. https://doi.org/10.1016/j.cis.2021.102584.
6. Jung, Y. C.; Bhushan, B. Wetting Behavior of Water and Oil Droplets in Three-Phase Interfaces for Hydrophobicity/Philicity and Oleophobicity/Philicity. *Langmuir* **2009**, *25* (24), 14165–14173. https://doi.org/10.1021/la901906h.
7. Baier, R. E. Surface Behaviour of Biomaterials: The Theta Surface for Biocompatibility. *J Mater Sci Mater Med* **2006**, *17* (11), 1057–1062. https://doi.org/10.1007/s10856-006-0444-8.
8. Jin, H.; Tian, L.; Bing, W.; Zhao, J.; Ren, L. Bioinspired Marine Antifouling Coatings: Status, Prospects, and Future. *Prog Mater Sci* **2022**, *124* (November 2021), 100889. https://doi.org/10.1016/j.pmatsci.2021.100889.

9. (30) Brady, R. F.; Singer, I. L. Mechanical Factors Favoring Release from Fouling Release Coatings. *Biofouling* **2000**, *15* (1–3), 73–81. https://doi.org/10.1080/08927010009386299.
10. Koch, K.; Bhushan, B.; Barthlott, W. Multifunctional Surface Structures of Plants: An Inspiration for Biomimetics. *Prog Mater Sci* **2009**, *54* (2), 137–178. https://doi.org/10.1016/j.pmatsci.2008.07.003.
11. Ensikat, H. J.; Ditsche-Kuru, P.; Neinhuis, C.; Barthlott, W. Superhydrophobicity in Perfection: The Outstanding Properties of the Lotus Leaf. *Beilstein Journal of Nanotechnology* **2011**, *2* (1), 152–161. https://doi.org/10.3762/bjnano.2.19.
12. Gao, X.; Yan, X.; Yao, X.; Xu, L.; Zhang, K.; Zhang, J.; Yang, B.; Jiang, L. The Dry-Style Antifogging Properties of Mosquito Compound Eyes and Artificial Analogues Prepared by Soft Lithography. *Advanced Materials* **2007**, *19* (17), 2213–2217. https://doi.org/10.1002/adma.200601946.
13. Darmanin, T.; Guittard, F. Superhydrophobic and Superoleophobic Properties in Nature. *Materials Today* **2015**, *18* (5), 273–285. https://doi.org/10.1016/j.mattod.2015.01.001.
14. Yong, J.; Chen, F.; Fang, Y.; Huo, J.; Yang, Q.; Zhang, J.; Bian, H.; Hou, X. Bioinspired Design of Underwater Superaerophobic and Superaerophilic Surfaces by Femtosecond Laser Ablation for Anti- or Capturing Bubbles. *ACS Appl Mater Interfaces* **2017**, *9* (45), 39863–39871. https://doi.org/10.1021/acsami.7b14819.
15. Yong, J.; Chen, F.; Huo, J.; Fang, Y.; Yang, Q.; Bian, H.; Li, W.; Wei, Y.; Dai, Y.; Hou, X. Green, Biodegradable, Underwater Superoleophobic Wood Sheet for Efficient Oil/Water Separation. *ACS Omega* **2018**, *3* (2), 1395–1402. https://doi.org/10.1021/acsomega.7b02064.
16. Keller, N.; Bruchmann, J.; Sollich, T.; Richter, C.; Thelen, R.; Kotz, F.; Schwartz, T.; Helmer, D.; Rapp, B. E. Study of Biofilm Growth on Slippery Liquid-Infused Porous Surfaces Made from Fluoropor. *ACS Appl Mater Interfaces* **2019**, *11* (4), 4480–4487. https://doi.org/10.1021/acsami.8b12542.
17. Helmer, D.; Keller, N.; Kotz, F.; Stolz, F.; Greiner, C.; Nargang, T. M.; Sachsenheimer, K.; Rapp, B. E. Transparent, Abrasion-Insensitive Superhydrophobic Coatings for Real-World Applications. *Sci Rep* **2017**, *7* (1), 1–6. https://doi.org/10.1038/s41598-017-15287-8.
18. Das, S.; Kumar, S.; Samal, S. K.; Mohanty, S.; Nayak, S. K. A Review on Superhydrophobic Polymer Nanocoatings: Recent Development and Applications. *Ind Eng Chem Res* **2018**, *57* (8), 2727–2745. https://doi.org/10.1021/acs.iecr.7b04887.
19. Nimittrakoolchai, O. U.; Supothina, S. Deposition of Organic-Based Superhydrophobic Films for Anti-Adhesion and Self-Cleaning Applications. *J Eur Ceram Soc* **2008**, *28* (5), 947–952. https://doi.org/10.1016/j.jeurceramsoc.2007.09.025.

20. Ding, X.; Zhou, S.; Gu, G.; Wu, L. A Facile and Large-Area Fabrication Method of Superhydrophobic Self-Cleaning Fluorinated Polysiloxane/TiO2 Nanocomposite Coatings with Long-Term Durability. *J Mater Chem* **2011**, *21* (17), 6161–6164. https://doi.org/10.1039/c0jm04546b.
21. (Sas, I.; Gorga, R. E.; Joines, J. A.; Thoney, K. A. Literature Review on Superhydrophobic Self-Cleaning Surfaces Produced by Electrospinning. *J Polym Sci B Polym Phys* **2012**, *50* (12), 824–845. https://doi.org/10.1002/polb.23070.
22. Drelich, J.; Chibowski, E.; Meng, D. D.; Terpilowski, K. Hydrophilic and Superhydrophilic Surfaces and Materials. *Soft Matter* **2011**, *7* (21), 9804–9828. https://doi.org/10.1039/c1sm05849e.
23. RN, W. Engineering Chemistry. *Ind Eng Chem Res* **1936**, *28*, 988–994. https://doi.org/10.1017/cbo9781316146743.
24. Otitoju, T. A.; Ahmad, A. L.; Ooi, B. S. Superhydrophilic (Superwetting) Surfaces: A Review on Fabrication and Application. *Journal of Industrial and Engineering Chemistry* **2017**, *47*, 19–40. https://doi.org/10.1016/j.jiec.2016.12.016.
25. Wei, Q.; Becherer, T.; Angioletti-Uberti, S.; Dzubiella, J.; Wischke, C.; Neffe, A. T.; Lendlein, A.; Ballauff, M.; Haag, R. Protein Interactions with Polymer Coatings and Biomaterials. *Angewandte Chemie - International Edition* **2014**, *53* (31), 8004–8031. https://doi.org/10.1002/anie.201400546.
26. Schlenoff, J. B. Zwitteration: Coating Surfaces with Zwitterionic Functionality to Reduce Nonspecific Adsorption. *Langmuir* **2014**, *30* (32), 9625–9636. https://doi.org/10.1021/la500057j.
27. Grozea, C. M.; Walker, G. C. Approaches in Designing Non-Toxic Polymer Surfaces to Deter Marine Biofouling. *Soft Matter* **2009**, *5* (21), 4088–4100. https://doi.org/10.1039/b910899h.
28. (49) Rosenhahn, A.; Sendra, G. H. Surface Sensing and Settlement Strategies of Marine Biofouling Organisms. *Biointerphases* **2012**, *7* (1–4), 1–13. https://doi.org/10.1007/s13758-012-0063-5.
29. Del Grosso, C. A.; Leng, C.; Zhang, K.; Hung, H. C.; Jiang, S.; Chen, Z.; Wilker, J. J. Surface Hydration for Antifouling and Bio-Adhesion. *Chem Sci* **2020**, *11* (38), 10367–10377. https://doi.org/10.1039/d0sc03690k.
30. Miller, D. J.; Dreyer, D. R.; Bielawski, C. W.; Paul, D. R.; Freeman, B. D. Surface Modification of Water Purification Membranes. *Angewandte Chemie - International Edition* **2017**, *56* (17), 4662–4711. https://doi.org/10.1002/anie.201601509.
31. Camós Noguer, A.; Olsen, S. M.; Hvilsted, S.; Kiil, S. Long-Term Stability of PEG-Based Antifouling Surfaces in Seawater. *J Coat Technol Res* **2016**, *13* (4), 567–575. https://doi.org/10.1007/s11998-016-9801-9.

32. Liu, V. A.; Jastromb, W. E.; Bhatia, S. N. Engineering Protein and Cell Adhesivity Using PEO-Terminated Triblock Polymers. *J Biomed Mater Res* **2002**, *60* (1), 126–134. https://doi.org/10.1002/jbm.10005.
33. Chen, S.; Li, L.; Zhao, C.; Zheng, J. Surface Hydration: Principles and Applications toward Low-Fouling/Nonfouling Biomaterials. *Polymer (Guildf)* **2010**, *51* (23), 5283–5293. https://doi.org/10.1016/j.polymer.2010.08.022.
34. Lee, J. H.; Lee, H. B.; Andrade, J. D. Blood Compatibility of Polyethylene Oxide Surfaces. *Prog Polym Sci* **1995**, *20* (6), 1043–1079. https://doi.org/10.1016/0079-6700(95)00011-4.
35. Shih, Y. J.; Chang, Y.; Quemener, D.; Yang, H. S.; Jhong, J. F.; Ho, F. M.; Higuchi, A.; Chang, Y. Y. Hemocompatibility of Polyampholyte Copolymers with Well-Defined Charge Bias in Human Blood. *Langmuir* **2014**, *30* (22), 6489–6496. https://doi.org/10.1021/la5015779.
36. Yang, W. J.; Neoh, K. G.; Kang, E. T.; Teo, S. L. M.; Rittschof, D. Polymer Brush Coatings for Combating Marine Biofouling. *Prog Polym Sci* **2014**, *39* (5), 1017–1042. https://doi.org/10.1016/j.progpolymsci.2014.02.002.
37. Ostuni, E.; Chapman, R. G.; Holmlin, R. E.; Takayama, S.; Whitesides, G. M. A Survey of Structure-Property Relationships of Surfaces That Resist the Adsorption of Protein. *Langmuir* **2001**, *17* (18), 5605–5620. https://doi.org/10.1021/la010384m.
38. Jeon, S. I.; Lee, J. H.; Andrade, J. D.; De Gennes, P. G. Protein-Surface Interactions in the Presence of Polyethylene Oxide. I. Simplified Theory. *J Colloid Interface Sci* **1991**, *142* (1), 149–158. https://doi.org/10.1016/0021-9797(91)90043-8.
39. Magin, C. M.; Finlay, J. A.; Clay, G.; Callow, M. E.; Callow, J. A.; Brennan, A. B. Antifouling Performance of Cross-Linked Hydrogels: Refinement of an Attachment Model. *Biomacromolecules* **2011**, *12* (4), 915–922. https://doi.org/10.1021/bm101229v.
40. Lundberg, P.; Bruin, A.; Klijnstra, J. W.; Nyström, A. M.; Johansson, M.; Malkoch, M.; Hult, A. Poly(Ethylene Glycol)-Based Thiol-Ene Hydrogel Coatings?Curing Chemistry, Aqueous Stability, and Potential Marine Antifouling Applications. *ACS Appl Mater Interfaces* **2010**, *2* (3), 903–912. https://doi.org/10.1021/am900875g.
41. Browning, M. B.; Cereceres, S. N.; Luong, P. T.; Cosgriff-Hernandez, E. M. Determination of the in Vivo Degradation Mechanism of PEGDA Hydrogels. *J Biomed Mater Res A* **2014**, *102* (12), 4244–4251. https://doi.org/10.1002/jbm.a.35096.
42. Ekblad, T.; Bergström, G.; Ederth, T.; Conlan, S. L.; Mutton, R.; Clare, A. S.; Wang, S.; Liu, Y.; Zhao, Q.; D'Souza, F.; Donnelly, G. T.; Willemsen, P. R.; Pettitt, M. E.; Callow, M. E.; Callow, J. A.; Liedberg, B. Poly(Ethylene Glycol)-Containing Hydrogel Surfaces for Antifouling Applications in Marine and Freshwater Environments. *Biomacromolecules* **2008**, *9* (10), 2775–2783. https://doi.org/10.1021/bm800547m.

43. Schmidt, D. L.; Brady, R. F.; Lam, K.; Schmidt, D. C.; Chaudhury, M. K. Contact Angle Hysteresis, Adhesion, and Marine Biofouling. *Langmuir* **2004**, *20* (7), 2830–2836. https://doi.org/10.1021/la035385o.
44. Ujjain, S. K.; Roy, P. K.; Kumar, S.; Singha, S.; Khare, K. Uniting Superhydrophobic, Superoleophobic and Lubricant Infused Slippery Behavior on Copper Oxide Nano-Structured Substrates. *Sci Rep* **2016**, *6* (June), 1–10. https://doi.org/10.1038/srep35524.
45. Wu, C. J.; Li, Y. F.; Woon, W. Y.; Sheng, Y. J.; Tsao, H. K. Contact Angle Hysteresis on Graphene Surfaces and Hysteresis-Free Behavior on Oil-Infused Graphite Surfaces. *Appl Surf Sci* **2016**, *385*, 153–161. https://doi.org/10.1016/j.apsusc.2016.05.059.
46. Wong, T. S.; Kang, S. H.; Tang, S. K. Y.; Smythe, E. J.; Hatton, B. D.; Grinthal, A.; Aizenberg, J. Bioinspired Self-Repairing Slippery Surfaces with Pressure-Stable Omniphobicity. *Nature* **2011**, *477* (7365), 443–447. https://doi.org/10.1038/nature10447.
47. Ba, M.; Zhang, Z.; Qi, Y. Fouling Release Coatings Based on Polydimethylsiloxane with the Incorporation of Phenylmethylsilicone Oil. *Coatings* **2018**, *8* (5). https://doi.org/10.3390/coatings8050153.
48. Amini, S.; Kolle, S.; Petrone, L.; Ahanotu, O.; Sunny, S.; Sutanto, C. N.; Hoon, S.; Cohen, L.; Weaver, J. C.; Aizenberg, J.; Vogel, N.; Miserez, A. Preventing Mussel Adhesion Using Lubricant-Infused Materials. *Science (1979)* **2017**, *357* (6352), 668–673. https://doi.org/10.1126/science.aai8977.
49. Galhenage, T. P.; Hoffman, D.; Silbert, S. D.; Stafslien, S. J.; Daniels, J.; Miljkovic, T.; Finlay, J. A.; Franco, S. C.; Clare, A. S.; Nedved, B. T.; Hadfield, M. G.; Wendt, D. E.; Waltz, G.; Brewer, L.; Teo, S. L. M.; Lim, C. S.; Webster, D. C. Fouling-Release Performance of Silicone Oil-Modified Siloxane-Polyurethane Coatings. *ACS Appl Mater Interfaces* **2016**, *8* (42), 29025–29036. https://doi.org/10.1021/acsami.6b09484.
50. Zhang, H.; Liang, Y.; Wang, P.; Zhang, D. Design of Slippery Organogel Layer with Room-Temperature Self-Healing Property for Marine Anti-Fouling Application. *Prog Org Coat* **2019**, *132* (February), 132–138. https://doi.org/10.1016/j.porgcoat.2019.03.020.
51. He, X.; Lou, T.; Yang, Z.; Bai, X.; Yuan, C.; Wang, C.; Neville, A. Lubricant-Infused Titania Surfaces with High Underwater Transparency for Antifouling Applications: A Combined Experimental and Molecular Dynamics Study. *Appl Surf Sci* **2021**, *543* (December 2020), 148848. https://doi.org/10.1016/j.apsusc.2020.148848.
52. Togasawa, R.; Tenjimbayashi, M.; Matsubayashi, T.; Moriya, T.; Manabe, K.; Shiratori, S. A Fluorine-Free Slippery Surface with Hot Water Repellency and Improved Stability against Boiling. *ACS Appl Mater Interfaces* **2018**, *10* (4), 4198–4205. https://doi.org/10.1021/acsami.7b15689.

53. Chen, K.; Zhou, S.; Wu, L. Self-Healing Underwater Superoleophobic and Antibiofouling Coatings Based on the Assembly of Hierarchical Microgel Spheres. *ACS Nano* **2016**, *10* (1), 1386–1394. https://doi.org/10.1021/acsnano.5b06816.
54. Ou, X.; Cai, J.; Tian, J.; Luo, B.; Liu, M. Superamphiphobic Surfaces with Self-Cleaning and Antifouling Properties by Functionalized Chitin Nanocrystals. *ACS Sustain Chem Eng* **2020**, *8* (17), 6690–6699. https://doi.org/10.1021/acssuschemeng.0c00340.
55. Li, J.; Han, X.; Li, W.; Yang, L.; Li, X.; Wang, L. Progress in Materials Science Nature-Inspired Reentrant Surfaces. **2023**, *133* (February 2022), 1–39.
56. Tuteja, A.; Choi, W.; Mabry, J. M.; McKinley, G. H.; Cohen, R. E. Designing Super-Oleophobic Surfaces with Fluoroposs. *2007 AIChE Annual Meeting* **2007**, No. December, 1618–1622.
57. Huang, X.; Mutlu, H.; Theato, P. A Bioinspired Hierarchical Underwater Superoleophobic Surface with Reversible PH Response. *Adv Mater Interfaces* **2020**, *7* (8), 1–7. https://doi.org/10.1002/admi.202000101.
58. Kobayashi, M.; Terayama, Y.; Yamaguchi, H.; Terada, M.; Murakami, D.; Ishihara, K.; Takahara, A. Wettability and Antifouling Behavior on the Surfaces of Superhydrophilic Polymer Brushes. *Langmuir* **2012**, *28* (18), 7212–7222. https://doi.org/10.1021/la301033h.
59. Szapoczka, W. K.; Larsen, V. H.; Böpple, H.; Kleinegris, D. M. M.; Diao, Z.; Skodvin, T.; Spatz, J. P.; Holst, B.; Thomas, P. J. Transparent, Antibiofouling Window Obtained with Surface Nanostructuring. *ACS Omega* **2024**. https://doi.org/10.1021/acsomega.4c03030.
60. Akhtar, N.; Thomas, P. J.; Svardal, B.; Almenningen, S.; De Jong, E.; Magnussen, S.; Onck, P. R.; Fernø, M. A.; Holst, B. Pillars or Pancakes? Self-Cleaning Surfaces without Coating. *Nano Lett* **2018**, *18* (12), 7509–7514. https://doi.org/10.1021/acs.nanolett.8b02982.
61. Weber, F.; Esmaeili, N. Marine Biofouling and the Role of Biocidal Coatings in Balancing Environmental Impacts. *Biofouling* **2023**, *39* (6), 661–681. https://doi.org/10.1080/08927014.2023.2246906.
